# Mobile phone reminders and peer counseling improve adherence and treatment outcomes of patients on ART in Malaysia: A randomized clinical trial

**DOI:** 10.1371/journal.pone.0177698

**Published:** 2017-05-16

**Authors:** Surajudeen Abiola Abdulrahman, Lekhraj Rampal, Faisal Ibrahim, Anuradha P. Radhakrishnan, Hayati Kadir Shahar, Norlijah Othman

**Affiliations:** 1 Department of Community Health, Faculty of Medicine and Health Sciences, Universiti Putra Malaysia, UPM Serdang, Selangor Darul Ehsan, Malaysia; 2 Department of Public Health Medicine, Penang Medical College, George Town, Penang, Malaysia; 3 Infectious Disease Clinic, Hospital Sungai Buloh, Sungai Buloh, Selangor Darul Ehsan, Malaysia; 4 Department of Pediatrics, Faculty of Medicine and Health Sciences, Universiti Putra Malaysia, UPM Serdang, Selangor Darul Ehsan, Malaysia; Johns Hopkins School of Medicine, UNITED STATES

## Abstract

**Background:**

Adherence to treatment remains the cornerstone of long term viral suppression and successful treatment outcomes among patients receiving Antiretroviral Therapy (ART).

**Objective(s):**

Evaluate the effectiveness of mobile phone reminders and peer counseling in improving adherence and treatment outcomes among HIV positive patients on ART in Malaysia.

**Methods:**

A single-blind, parallel group RCT conducted in Hospital Sungai Buloh, Malaysia in which 242 adult Malaysian patients were randomized to intervention or control groups. Intervention consisted of a reminder module delivered through SMS and telephone call reminders by trained research assistants for 24 consecutive weeks (starting from date of ART initiation), in addition to adherence counseling at every clinic visit. The length of intended follow up for each patient was 6 months. Data on adherence behavior of patients was collected using specialized, pre-validated Adult AIDS Clinical Trial Group (AACTG) adherence questionnaires. Data on weight, clinical symptoms, CD4 count and viral load tests were also collected. Data was analyzed using SPSS version 22 and R software. Repeated measures ANOVA, Friedman’s ANOVA and Multivariate regression models were used to evaluate efficacy of the intervention.

**Results:**

The response rate after 6 months follow up was 93%. There were no significant differences at baseline in gender, employment status, income distribution and residential location of respondents between the intervention and control group. After 6 months follow up, the mean adherence was significantly higher in the intervention group (95.7; 95% CI: 94.39–96.97) as compared to the control group (87.5; 95% CI: 86.14–88.81). The proportion of respondents who had Good (>95%) adherence was significantly higher in the intervention group (92.2%) compared to the control group (54.6%). A significantly lower frequency in missed appointments (14.0% vs 35.5%) (p = 0.001), lower viral load (p = 0.001), higher rise in CD4 count (p = 0.017), lower incidence of tuberculosis (p = 0.001) and OIs (p = 0.001) at 6 months follow up, was observed among patients in the intervention group.

**Conclusion:**

Mobile phone reminders (SMS and telephone call reminders) and peer counseling are effective in improving adherence and treatment outcomes among HIV positive patients on ART in Malaysia. These findings may be of potential benefit for collaborative adherence planning between patients and health care providers at ART commencement.

## Introduction

In Malaysia, by the end of 2013, there was a cumulative figure of 101, 672 reported HIV cases, 20, 235 AIDS cases, 16, 340 deaths and 85, 332 living with HIV and 3, 393 new infections [1]. Adherence to medication remains the cornerstone of long-term HIV suppression [2]. Adherence to Antiretroviral (ARV) medications prevents disease progression, and the emergence of resistant mutations, thereby reducing morbidity, and the necessity for more frequent, complicated regimens which are also relatively more expensive [3]. Several studies have prescribed a minimum adherence level of 95% for treatment success [4–7], other studies have demonstrated that ARV medication adherence levels of 54–95% is required to maintain prolonged viral load suppression depending on the allowable flexibility margins of each Antiretroviral Therapy (ART) program [2].

The gap between the number of those requiring ART and those who have access to it still leaves much to be desired, with most developing countries still having 50%–60% unmet needs. The Malaysian Government almost entirely provides all the funds for HIV treatment care and support for about 17,369 patients at no cost to the patients on first line medications and heavily subsidizes for those on second line ARVs [1]. The current number of patients on ART in Malaysia represents only about 47% of the estimated number of PLHIV eligible for ART (37, 274). Stigma and discrimination as well as poor adherence to treatment (clinic visits and medication adherence) remain a stumbling block to the success of treatment programs. Poor adherence is also a significant cause of treatment failure, disease progression and death among HIV patients. It also has grave socioeconomic impact on program funding, as more patients who fail on first line regimens have to be provided with the more expensive and complex second line medications [3]. Recent innovations using mobile phone technologies such as text messaging to improve medication adherence among patients on ART have been examined and implemented across many countries with high quality evidence suggesting the efficacy of weekly SMS reminders to patients in improving adherence to ART when compared to standard care [8–11]. However, further studies across low, middle and high income countries, scale-up of program evidence in hospitals, including cost-effectiveness analysis were recommended. Research evidence has also shown that real-time adherence monitoring and intervention through the use of medication storage devices equipped with mobile phone technology is now possible. Sabin et al, 2015 [12] demonstrated among ART cohort in China that triggered, real-time cell phone reminders in addition to counseling, significantly improved adherence behavior of patients who received such intervention.

The objective of this study was to determine the effectiveness of mobile phone reminders (SMS and telephone call reminders) and peer counselling in improving adherence and treatment outcomes among HIV positive patients on ART in Malaysia.

## Materials and methods

### Study design and setting

This was a single-blind parallel group randomized controlled clinical trial in which patients initiating ART based on WHO 2013 guidelines underwent simple complete randomization to either standard care or weekly medication reminder SMS and telephone call reminders for scheduled clinic appointments. The study was conducted in Hospital Sungai Buloh, a semi-urban based, Government-owned referral hospital which provides comprehensive HIV prevention, care and treatment services to over 6,000 (about one-third) HIV/AIDS patients in Malaysia. Recruitment commenced in January 2014 and follow up ended in December 2014.

### Study procedures

Eligible adult HIV positive patients who had completed four weeks of vitamin training and were newly initiating ART based on 2013 WHO guidelines, with valid telephone numbers and able to read text messages were included in the study and randomized to either of two treatment arms based on simple, complete randomization technique in a ratio 1:1 for intervention and control groups, respectively. Based on a priori decision in our trial protocol aimed at maximizing power and efficiency of the study, we generated from a random number program, equal sets of unique numbers based on which group balance in random allocation was achieved. Those excluded from the study include all HIV positive patients already commenced (current) or restarting ART due to previous default and/or lost-to-follow-up (LTFU) status, pregnant patients receiving ART and foreigners. Patients were enrolled if they understood and consented for randomization to either standard care (routine adherence counseling and paper-based appointment scheduling) or weekly medication reminder SMS and telephone call reminders for scheduled clinic appointments as well as peer counseling during clinic visits.

Once enrolled and randomized to either of the treatment arms, baseline data on socio-demographic factors, clinical symptoms and adherence behavior of respondents was collected using modified, pre-validated, self-administered Adult AIDS Clinical Trial Group (AACTG) adherence questionnaires. A “Reminder module” which included standardized weekly SMS medication reminders (sent at 9am every Monday); SMS reminder 3 days prior to scheduled clinic appointments (individualized and sent at lunch time), and an average of 90sec lunch hour telephone call reminders a day prior to scheduled clinic appointment (in addition to standard care—routine adherence counseling and paper-based appointment scheduling) was delivered consistently for 24 consecutive weeks (starting from date of ART initiation and baseline data collection) to respondents in the intervention group by two trained PLHIV (research assistants) while respondents in the control group received standard care only. Each patient in the intervention group had a minimum of three (during clinic visits at month 1, month 3 and month 6) individual counselling sessions with the research assistants lasting an average of 15 minutes per encounter. To ensure confidentiality, typical medication reminder text messages included a short slogan in Malay language “Apa khabar” “Ini untuk menberithau anda ubat” meaning “How are you?” “This is to remind you of your medications”. Appointment reminder text message was “Apa khabar” “Tolong ingat tarikh temu janji lusa” meaning “How are you?” “Remember your appointment day after tomorrow” and telephone conversation was standardized and short, with the message “Apa khabar” “Tolong ingat tarikh temu janji besok” meaning “How are you?” “Remember your appointment tomorrow”. Patients were not required to provide any responses to the text messages. However, a log of text message communications and telephone calls was recorded and kept. Typically, clinic visits were scheduled by clinicians in the study setting at ART initiation (visit 1), 2 weeks after ART initiation (visit 2), 1 month (visit 3), 3 months (visit 4) and 6 months after ART initiation (visit 5). Within this period, every outpatient clinic attendance were planned such that patients are clinically assessed by a clinician, receive adherence counseling from a nurse or doctor, perform or receive feedback on their laboratory investigation (if required) and pick up their Anti-retroviral medications. Prior to this study, standardized mechanisms for defaulter tracking were not in place in the study location.

### Outcome measures

At study commencement, baseline medication adherence was estimated based on self-reported adherence to a mandatory vitamin training course in the preceding four weeks period, using specialized AACTG baseline adherence questionnaires. As a standard best practice in the study setting, only those who were clinically assessed as being sufficiently adherent to the vitamin training were eligible to commence ART. Thereafter, adherence to ART was measured at 3 and 6 months post-ART initiation using specialized AACTG follow-up adherence questionnaires. Adherence was measured on a continuous scale ranging from 0 to 100, and scores were calculated using a standardized adherence index formula adopted from Reynolds et al. [13] which took into account self-reported adherence to prescribed medication doses (number of missed drug doses and period of missed medications), dietary and other instructions in the 4-day period and weekend preceding clinical visit and assessment. Individual respondent scores were computed using the following formula:

Adherence=100×{[(0.65+(2.15×Adhwk1)+(2.21×Adhwk2)+(2.07×Adhwk3)+(1.99×Adhwk4)+(0.37×Follwsch)+(0.36×Instfu)−0.13×Lastskip)]÷11.99}

Adherence scores were further categorized into two levels based on WHO 2005 recommendations, and included (a) Optimal (Good) adherence–a patient who reportedly misses <3 doses of drugs per month, considered as >95% adherence (b) Sub-optimal (comprising Fair and Poor) adherence–a patient who reportedly misses 3 or more doses of drugs per month, considered ≤95% adherence (WHO, 2005).

Data on regularity of respondents’ scheduled clinic visits was obtained by the research assistants (for whom access was duly sought and provided by the hospital management) from the hospital’s electronic medical records system using standardized data extraction forms and corroborated with drug refill appointments from pharmacy records. Outpatient clinic attendance was measured based on number of outpatient clinic appointments attended on-schedule and number of missed appointments. Regularity of scheduled outpatient clinic attendance was then categorized into two levels, which included (a) Regular attendee–a person who has never missed any scheduled clinic appointment (b) Defaulter–a person who has missed one or more scheduled clinic appointment, for any reason(s). Patients were considered to be lost to follow up if s/he refuses to show up for scheduled clinic visit for 3 consecutive months, after 3 consecutive attempts to track the client and bring them back on treatment. Only attendance records for five consecutive scheduled outpatient clinic visits within the first 6 months of ART initiation were considered. Visits to emergency room, inpatient ward admissions, unscheduled, changed or cancelled outpatient clinic appointments were not considered in this study. Clinic attendance was considered valid irrespective of the time of visit on the scheduled appointment day.

Results of baseline CD4 count, viral load, full blood count, liver function and renal profile of respondents were retrieved from laboratory records onto the data extraction forms. The tests were conducted an average of five weeks prior to ART initiation/study commencement, and served as the basis for the clinician’s decision on ART eligibility and start of vitamin training for the respondents. These tests were further repeated at 6 months as part of the standard follow-up procedures in the study setting.

The research assistants accessed and recorded onto the data extraction forms, baseline and 6 months’ follow-up information on patient’s blood pressure, TB status, opportunistic infection (OI) index and body weight from clinicians’ notes in the electronic medical record system. TB status consisted of both clinical and microbiological evaluation, while OI index was determined by clinicians based on presence/absence WHO clinical stage-defining illnesses. Data extraction forms were reviewed periodically for completeness, correctness and accuracy by the site study coordinator.

### Randomization and blinding

A computer-based randomization sequence was generated by an independent biostatistician using Stata 11.0 statistical software. After consenting to participate (by filling and signing a consent form) and meeting the inclusion criteria, screened subjects were enrolled and immediately afterwards were assigned to a randomized study arm by the study coordinator opening the sealed envelopes to determine allocation. Written allocation of assignment was sealed in individual opaque envelopes marked with study identification numbers which was made available in the study clinic to allocate the target number of participants.

The principal investigator and data analyst were blinded to the study arm. The ART clinician, nurse counselors and other members of the treatment team were unaware of the randomization assignment during enrolment and follow up period. Due to the nature of the study, it was impractical to blind participants post-randomization. However, the participants were strictly instructed not to mention their group assignment or any text messages to these staff. Allocation concealment, blinding and standardized training of the research assistants on research ethics and confidentiality was conducted to minimize the risk of cross-contamination.

### Sample size

To detect a 20% increase in medication adherence from baseline values over a 6-months period, we determined using formula for calculating sample size in hypothesis testing by comparing two means [14], that a total of 108 patients (59 per study arm) including adjustment for 10% attrition, would provide at least 80% power assuming a two-tailed test and type 1 error rate of 5%. Compared to other similar studies, we observed that this sample size was too small and may result in poor precision of our study estimates. Therefore, we repeated the sample size calculation using the secondary outcomes of the study. We observed that to detect a 14% increase in CD4 count from baseline values over a 6-months period, 220 patients (110 per study arm) would provide at least 80% power assuming a two-tailed test and type 1 error rate of 5%. We increased the sample size to 242 to account for approximately 10% attrition.

### Study outcome

The primary outcome of the study was adherence (improved scheduled clinic attendance and medication adherence self-report), while secondary outcomes were Immunological (CD4 count) Virological (Viral load), and Clinical (Weight, TB status and OI index).

### Statistical analysis

Data collected was checked, cleaned, entered into and analysed using Statistical Package for Social Sciences software (SPSS) version 22 (IBM, 2014) and R statistical software (R/R Studio, GNU Project, 2014). Primary analysis was conducted on an intent-to-treat basis and included all the patients. Parametric tests (T-test, 2-way repeated measures ANOVA) and non-parametric tests (Chi square test, Fisher’s exact test, Friedman’s ANOVA) were conducted on the data. We adjusted for group differences in baseline characteristics such as age, ethnicity and education level using multivariate analysis.

### Ethical statement

This trial was retrospectively registered with ClinicalTrials.gov, NCT02677675, owing to limited understanding of ICMJE’s mandatory requirement of prospective trial registration in an approved registry by the authors who performed this experiment. The authors erroneously believed that, like most other clinical trials in Malaysia, prior registration with National Medical Research Register sufficed. The Universiti Putra Malaysia (UPM) Ethics committee for Human Research at its meeting on 6^th^ September, 2013 (UPM/TNCPI/RMC/1.4.18.1 (JKEUPM)/F1) and the Malaysian Ministry of Health’s Institutional Review and Ethics Committee (23^rd^ December, 2013) approved the study protocol. Prior to the enrolment of participants, the trial was registered in the National Medical Research Register (NMRR-13-882-17412), National Institutes of Health, Ministry of Health Malaysia. All study participants provided written informed consent. The first patient was recruited on 3^rd^ January, 2014 and the last of 242 participants on 2^nd^ July, 2014. The length of intended follow up for each individual patient was 6 months. Follow-up ended for all study participants on 22^nd^ December, 2014. The authors confirm that all on-going and related trials for this intervention are registered.

## Results

### Population characteristics

**Fig 1:**
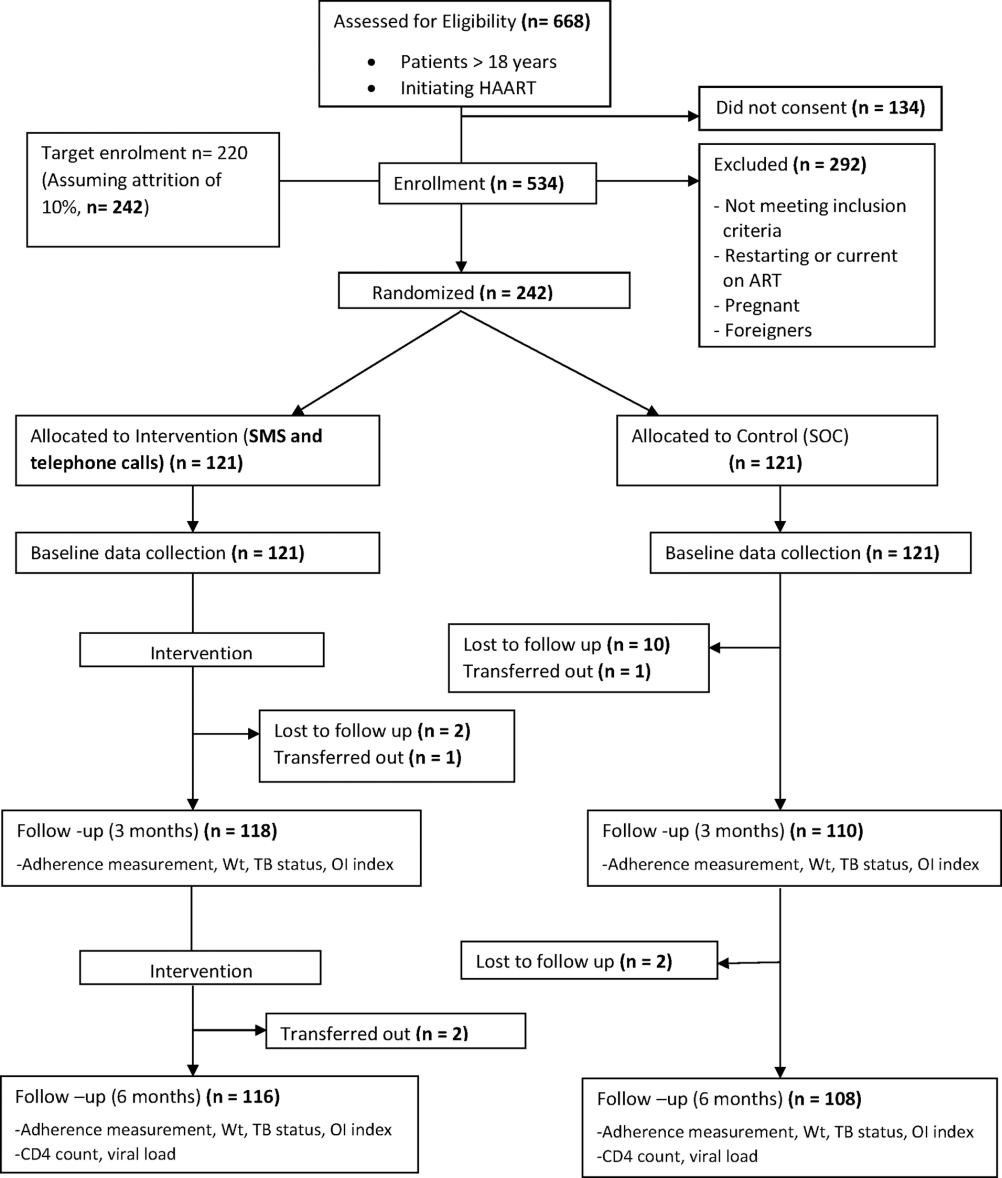
Consort flow diagram of the trial.

Majority (93%) of respondents completed six month follow up assessment (Fig 1). The overall mean age of 242 respondents was 33.4 ± 9.2 SD (95% CI: 32.23–34.56) years and ranged from 18 to 64 years. Majority (88.8%; 215/242) were males. There was no significant difference in gender (p = .838), income distribution (p = .071), the employment distribution (p = .473) and residential location (p = .544) between the intervention and control group at baseline (Table 1). However, Table 1 also shows statistically significant differences in age (p = .031), ethnicity (p = .036) and education level (p = .004) between the intervention and control group respondents at baseline. These undesired differences were controlled for in subsequent multivariate analyses.

**Table 1:** Baseline socio-demographic characteristics of respondents.

Characteristics	Intervention group (n = 121)	Control group (n = 121)	P value
	n (%)	n (%)	
**Gender**			
Male	107 (88.4)	108 (89.3)	0.838^a^
Female	14 (11.6)	14 (10.7)	
**Ethnicity**			
Malay	64 (52.9)	53 (43.8)	0.036^b^
Chinese	39 (32.2)	59 (48.8)	
Indian	14 (11.6)	8 (6.6)	
Others	4 (3.3)	1 (0.8)	
**Education level**			
Less than Bachelor degree	71 (58.7)	93 (76.9)	0.004^a^
Bachelor degree and above	50 (41.3)	28 (23.1)	
**Monthly Income (RM)**			
<1500	21 (17.4)	39 (32.2)	0.071^a^
1500–2499	29 (24.0)	28 (23.1)	
2500–3499	32 (26.4)	29 (24.0)	
3500–4999	18 (14.8)	12 (9.9)	
>5000	21 (17.4)	13 (10.7)	
**Employment Status**			
Unemployed	13 (10.7)	18 (14.9)	0.473^a^
Self-employed	19 (15.7)	17 (14.0)	
Government employee	14 (11.6)	7 (5.8)	
Private organization employee	70 (57.9)	75 (61.9)	
Others	5 (4.1)	4 (3.3)	
**Residential Location**			
Rural	11 (9.1)	16 (13.2)	0.544^a^
Semi-urban	43 (35.5)	44 (36.4)	
Urban	67 (55.4)	61 (50.4)	
**Age (years), mean ± SD**	32.1 ± 8.7	34.7 ± 9.5	0.031^c^

^a^P value obtained by chi-square test.

^b^P value obtained by Fisher’s exact test (FET).

^c^P value obtained by student t- test for independent samples.

RM = Ringgit Malaysia

There was no statistically significant group differences in distribution of HAART regimen (p = 1.000) among respondents at baseline (Table 2). We also observed no statistically significant group differences in mean CD4 count (p = .297), viral load (p = .144), weight (p = .955) and adherence (p = .212) at baseline (Table 2).

**Table 2:** Baseline regimen, CD4 count, viral load, weight and medication adherence.

Variables	Intervention group (n = 121)	Control group (n = 121)	P value
	n (%)	n (%)	
**HAART Regimen**			
ZDV/3TC/NVP	2 (1.7)	5 (4.1)	1.000^a^
ZDV/3TC/EFV	59 (48.8)	57 (47.1)	
TDF/FTC/NVP	1 (0.8)	1 (0.8)	
TDF/FTC/EFV	58 (47.9)	56 (46.3)	
ZDV/3TC/RALTEGRAVIR	1 (0.8)	1 (0.8)	
D4T/3TC/EFV	0 (0.0)	1 (0.8)	
**Baseline medication adherence level**			
Poor adherence	28 (23.1)	20 (16.5)	0.197^b^
Good adherence	93 (76.9)	101 (83.5)	
	**mean ± SD**	**mean ± SD**	
**CD4 count (cells/μl)**	232.64 ± 137.9	213.31 ± 149.4	0.297^c^
**Viral Load (Log10)**	4.61 ± 0.9	4.85 ± 1.1	0.144^c^
**Weight (Kg)**	61.19 ± 11.4	61.47 ± 12.3	0.955^c^
**Mean adherence**	80.1 ± 19.8	85.1 ± 15.8	0.212^c^

SD, Standard Deviation.

^a^P value obtained by Fisher’s exact test.

^b^P value obtained by chi-square test.

^c^P value obtained by student t- test for independent samples.

100% of respondents in the intervention group received appointment reminder SMS, while 96% (457/476) of appointment reminder telephone calls were successfully completed. Although they were not mandated to provide a response to the SMS, 82% (99/121) of respondents replied the SMS at least once during the 24 week period. The most common response (94%) was “I’m fine, thank you for your reminder”. 6% (7/121) of the respondents replied the SMS by asking questions related to adverse effects of their medications or simply seeking appointment rescheduling.

In the course of follow up, 8 (7%) respondents in the intervention group and 7 (6%) in the control group had substitutions within first-line regimen. There were no regimen switches from first to second, third or salvage therapy in both groups during the follow up period.

### Effect of intervention on outcome measures

#### Adherence to ART

Mean adherence to ART increased from baseline values of 80.1 ± 19.6 to 95.7 ± 1.6 in the intervention group as compared to 85.1 ± 15.8 to 87.5 ± 9.9 in the control group after 6 months follow up. This difference in the mean adherence to ART between the two groups after 6 months follow up was statistically significant (p = .035) (Table 3). After six months follow up, the proportion of respondents who had Good adherence (>95%) was significantly higher in the intervention group (n = 107, 92.2%) as compared to control group (n = 59, 54.6%) (p = .001).

**Table 3:** Summary table of 2-way repeated measures ANOVA showing main effect of group, time and group x time interaction for adherence, CD4 count, viral load and weight.

Outcome measure	Baseline	6 months follow up	Group	Time	Group x time
**Adherence**	mean ±SD (95% CI)	mean ±SD (95% CI)			
Intervention	80.14 ± 19.6 (76.34–86.44)	95.68 ± 1.6 (94.39–96.97)	0.035*	0.001*	0.005*
Control	85.10 ± 15.8 (79.75–90.22)	87.47 ± 9.9 (86.14–88.81)			
**CD4 count**					
Intervention	234.62 ± 138.4 (207.93–261.31)	380.63 ± 193.9 (347.89–413.38)	0.017*	0.001*	0.001*
Control	211.57 ± 153.5 (183.91–239.24)	305.19 ± 161.3 (271.26–339.13)			
**Viral Load (Log10)**					
Intervention	4.61 ± 0.9 (4.44–4.78)	1.41 ± 0.2 (1.28–1.54)	0.001*	0.001*	0.491
Control	4.85 ± 0.9 (4.67–5.02)	1.76 ± 0.9 (1.63–1.89)			
**Weight**					
Intervention	61.23 ± 11.5 (59.03–63.43)	62.75 ± 12.2 (60.39–65.10)	0.653	0.001*	0.313
Control	61.46 ± 12.8 (59.16–63.76)	63.77 ± 13.7 (61.31–66.23)			

*Significant at P<0.05

SD, Standard Deviation; CI, Confidence Interval. Group x time interaction represents the treatment effect as the difference in change-from-baseline between the two treatment groups

#### CD4 count in the intervention and control group after six months follow up

A significantly higher rise in CD4 count (p = .017) was observed in the intervention group after six months follow up. CD4 count increased by 146.01 cells/μl in the intervention group whereas an increase of 93.62 cells/μl was observed in the control group (Table 3).

#### Viral load in the intervention and control group after six months follow up

A significantly higher viral load (F = 14.531, p = .001) was observed among respondents in the control group at six months follow up (Table 3). Respondents in the intervention group achieved viral load suppression up to 1.41 (log10) compared to 1.76 (log10) in the control group. We further categorized respondents viral load counts to determine number of respondents who achieved viral suppression or not. We defined viral suppression as viral load below detection (<400 copies/ml) after 6 months of ART (Bartlett and Garlant, 2004; Aidsmap, 2004). We found that 99.1% of 107 respondents in the intervention group who achieved Optimal/Good adherence (>95%) had viral suppression compared to 89.3% (n = 28) in the control group, p = .028 (FET).

#### Adjusted effect of intervention on outcomes after controlling for baseline group differences

A mixed repeated measures ANCOVA was conducted to adjust for the potential effects of baseline treatment group imbalance in age, ethnicity, education level and monthly income, on the study outcomes. We examined group x time interactions as the difference in change-from-baseline between the two treatment groups that could be accounted for by any particular factor or combination of factors. In all situations examined, there was no significant interaction effect between the outcome variables and any of the covariates (p>.05). The results in Table 4 showed that a significant difference in treatment effect on adherence (p = .002) and CD4 count (p = .001) over time was observed between the two treatment groups, despite adjusting for imbalance in age, ethnicity, education level and monthly income at baseline. Similarly, adjustment for these covariates did not significantly change the treatment effect on viral load (p = .576) and weight (p = .359) over time between the two treatment groups.

**Table 4:** Summary table of mixed repeated measures ANCOVA showing main effect of group, time and group x time interaction for adherence, CD4 count, viral load and weight.

Outcome Measure	Unadjusted			Adjusted^†^		
	Group	Time	Group x time	Group	Time	Group x time
**Adherence**	0.035*	0.001*	0.005*	0.338	0.229	0.002**
**CD4 count**	0.017*	0.001*	0.001*	0.178	0.001**	0.001**
**Viral Load (log 10)**	0.001*	0.001*	0.491	0.001**	0.001**	0.576
**Weight**	0.653	0.001*	0.313	0.535	0.700	0.359

* Significant at P<0.05

**Significant at P<0.013 (Bonferroni correction)

^†^Adjusted for age, education level, monthly income and ethnicity

NOTE: P>0.05 for all interaction terms between covariates and outcomes

Group x time interaction represents the treatment effect as the difference in change-from-baseline between the two treatment groups

Overall, the baseline imbalances in age, ethnicity, education level and monthly income did not differentially change the response of either treatment groups to the treatment given over time (Table 4).

#### Hospital attendance in the intervention and control group after six months follow up

A higher frequency in missed appointments (35.5% vs 14.0%) (p = .001) was observed in the control group after six months follow up. Among the intervention group, out of 116 respondents, 104 (86%) attended their clinic appointments regularly. In the control group, out of 108 respondents, 78 (64.5%) attended their clinic appointments regularly. Similarly, a higher loss to follow up rate was observed in the control group (9.9%) as compared to the intervention group (1.7%) (Table 5).

**Table 5:** Subgroup analysis showing change in adherence level, TB status, OI index and hospital attendance.

Outcome measure	Intervention				Control			
	Baseline	6 months follow up	Change (%)	P value	Baseline	6 months follow up	Change (%)	P value
**Adherence level**								
Good	93 (76.9)	107 (92.2)	14 (15.1)	0.001^a^	101 (83.5)	59 (54.6)	-42 (41.6)	0.001^a^
Fair	0 (0.0)	9 (7.8)	9 (100)		0 (0.0)	17 (15.7)	17 (100)	
Poor	28 (23.1)	0 (0.0)	-28 (100)		20 (16.5)	32 (29.6)	12 (60.0)	
**TB status**								
No signs & symptoms of TB	103 (85.1)	112 (96.6)	9 (8.7)	0.002^a^	96 (79.3)	84 (77.8)	-12 (12.5)	0.001^a^
TB Suspect	13 (10.7)	1 (0.9)	-12 (92.3)		20 (16.5)	14 (12.9)	-6 (30.0)	
Currently on TB treatment	5 (4.1)	3 (2.6)	-2 (40.0)		5 (4.1)	10 (9.3)	-5 (100)	
**OI Index**								
No signs and symptoms of any new OI	103 (85.1)	115 (99.1)	12 (11.7)	0.001^b^	97 (80.2)	97 (89.8)	0 (0.0)	0.003^b^
1 or more WHO stage 2 defining disease	1 (0.8)	1 (0.9)	0 (0.0)		1 (0.8)	0 (0.0)	-1 (100)	
1 or more WHO stage 3 defining disease	8 (6.6)	0 (0.0)	-8 (100)		5 (4.1)	3 (2.8)	-2 (40.0)	
1 or more WHO stage 4 defining disease	9 (7.4)	0 (0.0)	-9 (100)		18 (14.9)	8 (7.4)	-10 (55.6)	
**Hospital Attendance**								
Regular attendee	-	104 (86.0)	-	0.001^b^	-	78 (64.5)	-	0.001^b^
Defaulter	-	12 (9.9)	-		-	30 (24.8)	-	
Lost to follow up	-	2 (1.7)	-		-	12 (9.9)	-	
Referred out	-	3 (2.5)	-		-	1 (0.8)	-	

Values are n (%); OI: Opportunistic infection; TB: Tuberculosis

^a^P value obtained by chi-square test.

^b^P value obtained by Fisher’s exact test.

#### TB Status and OI index in the intervention and control group after six months follow up

A higher incidence of TB (p = .001) and OIs (p = .001) at six months follow up, was observed among respondents in the control group (Table 5). In the intervention group, 112 out of 116 (96.6%) respondents had no signs and symptoms of TB, as compared to 84 out of 108 (77.8%) respondents in the control group. Similarly, 115 out of 116 (99.1%) respondents in the intervention group had no signs and symptoms of any new OI, as compared to 97 out of 108 (89.8%) respondents in the control group (Table 5).

## Discussion

The current study utilized weekly medication reminder SMS and peer counselling as a vehicle to improve medication adherence and clinic attendance among respondents in the intervention group. Despite the systematic nature with which the random allocation process was executed and monitored, undesired group imbalances in age, ethnicity and education level were observed in this study. It is possible that the relatively small sample size of this study [15] and long period of recruitment (6 months) might have contributed to this. Given that both groups of respondents arose from the same study base of patients attending HIV care at the study location, we believe that statistical adjustment for these baseline variables provided a valid means of protecting against potential chance bias and also improve the validity of our estimates [16, 17]. To the extent that the group x time interaction effect of our treatment on the outcomes did not change significantly in the adjusted analysis, we believe that the observed variance in the outcomes can potentially be attributable to the treatment provided.

In this RCT, the intervention produced a 15.5 percentage points increase (small effect size) in mean adherence at six months follow up. This is one of the highest intervention effects reported by any similar study in a developing country or resource-limited setting [9, 10]. However, inconsistent results have been reported among HIV populations in the United States [13, 18] and Brazil [19].

Similarly, our intervention produced a significantly higher proportion of regular attendees and a corresponding lower proportion of missed appointments among respondents in the intervention group compared to the control group. These findings are fairly comparable to results from previous studies in Uganda [20] and Australia [21]. In the current study, the commonest reasons cited for missed appointments were forgetfulness, busy schedule and being out-station, respectively. These findings are consistent with the reports of a prospective study conducted in Uganda [22].

Overall, the effectiveness, efficacy and acceptability of mobile phone messaging as a reminder tool to improve adherence and clinic attendance among HIV positive patients receiving ART has been suggested in the current study and other similar studies highlighted above. Improved clinic attendance can be leveraged upon to improve medication adherence through regular drug refills, re-enforcement of adherence counselling (particularly by peers) and providing an opportunity for closer monitoring of antiretroviral treatment.

It is clear that irrespective of the type of HIV epidemic–concentrated or generalized, socio-demographic differences and diversity in the study populations from different researches, strong evidence showing significant impact on adherence improvement using telephone messaging intervention can be achieved. However, adequate care must be taken in the interpretation of the specific findings of each research in view of the inherent weaknesses in the respective study designs and generalizability to the local context.

The findings of the current and earlier studies allude to the compelling evidence of a significant positive relationship between Good or optimal adherence (>95%) and increase in CD4 cell count among HIV positive patients receiving ART. We found that participants who received the intervention were more likely to be adherent and also achieve an average of 30% increase in CD4 count from baseline values. This is consistent with findings of other studies that have reported higher likelihood of achieving improved immunological function with >95% adherence [23–25, 9]. Consequently, individual and program-level interventions that are designed and targeted to improve ARV medication adherence would invariably produce a positive impact on immunologic response to treatment.

We found in this study that adherence was a significant predictor of virologic outcomes among the study cohort. Patients who received intervention were more likely to be adherent (>95%) and achieve viral suppression (<400 copies/ml) after six months on ART. Interestingly, there appear to be conflicting evidence in literature regarding this relationship. Similar intervention studies conducted previously reported varying degrees of success in achieving viral suppression [9, 23] while others practically found no difference in viral suppression even with the intervention [13, 24, 26, 27]

Overall, our study reported higher proportion of patients achieving viral suppression than most of the aforementioned studies above. Given the independent effect of ART on viral suppression which explains why respondents in both intervention and control groups achieved varying rates of viral suppression after six months of ART, focused interventions aimed at addressing medication adherence among patients receiving ART are likely to achieve even greater impact in viral suppression.

Very few studies have been conducted to evaluate the relationship between medication adherence and weight of respondents on ART. This study failed to demonstrate any significant group differences in weight gain at six months. This is probably due to a shorter duration of follow up compared to previously reported similar studies in Kenya [26] and Rwanda [28] demonstrating positive effect of intervention on respondent’s weight. Perhaps, other reasons for the observed differences in the relationship between medication adherence and weight found in our study and the Kenyan study mentioned above may be explained by factors such as difference in genetic composition, nutrition, socio-economic factors and level of physical activity between the respective study populations. We believe that further researches are required to establish strong evidence regarding the association between medication adherence and weight among HIV positive patients receiving ART.

We found a significant decrease in the proportion of patients in the intervention group who were TB suspects (92%) and having 1 or more clinical signs/symptoms of WHO clinical stage 3 and 4 disease (100%) at six months follow up compared to baseline values. Although improvements in TB status and OI index was also recorded among respondents in the control group, the changes observed were relatively higher among respondents in the intervention group.

Summarily, based on Cohen 2 (1988) guidelines, our intervention produced a small effect size of about 16% for mean adherence and CD4 count respectively and 14% for viral load.

### Strengths

Being the first intervention study to report efficacy of mobile phone and peer counseling intervention in improving adherence and treatment outcomes among HIV positive patients receiving ART in Malaysia, our study established a strong, causal, temporal, biologically plausible, and significant association between our intervention and outcomes of the study. The current study also established coherence between epidemiological (adherence self-report) and laboratory correlates of adherence behavior (CD4 count and viral load) among patients receiving ART in Malaysia. Our study is also one of the very few intervention studies that have examined the relationship between regularity of scheduled clinic attendance and medication adherence as well as immunological (CD4 count) and virological outcomes of antiretroviral therapy. The positive effect of intervention reported in this study has significant implications for achieving epidemic control and guiding HIV/AIDS intervention, especially in low-to-middle income countries and concentrated epidemics like Malaysia.

### Limitations

The limitations of this study included being single-centered, and use of adherence self-report. These did not markedly limit generalizability as other biological correlates of adherence behavior were measured, to corroborate self-reports. In addition to the fact that self-report of adherence behavior is widely believed to over-estimate true adherence (due to social desirability bias), baseline adherence scores presented in this study might have been further exaggerated by estimating adherence behavior based on 4 weeks of vitamin training (with little or no side effects) and adherence preparation prior to ART initiation, and not necessarily ARVs. Notwithstanding, this situation at best reduces the chances or probability of over-estimating the ‘true’ treatment effect of our intervention. Short (6 months) follow-up period of the current study may have resulted in adherence estimates that represent the ‘best case scenario’ evidence and not necessarily an accurate estimate/prediction of long term medication adherence. A more appropriate estimate of TB risk than the one provided in this study would have provided better insights and opportunities to address potential exposures among the patients. Given that only those clinically assessed to be sufficiently adherent to the vitamin training course were initiated on ART, and hence included in this study, these results may not expressly suggest similar effects of intervention when applied to those deemed ineligible to commence ART on account of poor adherence to vitamin training or patients with previous ART experience (stopped treatment, LTFU, switched regimens etc), and in settings where ‘test and treat’ is being practiced.

## Conclusion

Our study suggests the efficacy of the use of cost-effective and acceptable solutions such as mobile phone text messaging as a reminder tool to keep patients engaged, thereby promoting their adherence to ART, as part of a package of adherence interventions which includes regular peer-led adherence counseling. The ubiquitous nature of mobile phones even among HIV positive patients from low to middle income countries provides an excellent platform for targeted health interventions, irrespective of the nature of the epidemic, whether concentrated or generalized. Since the success of ART programs is largely measured by retention on treatment, the potential effects of this intervention in tracking patient’s clinic attendance and ensuring that they are retained in care could be valuable in HIV programming.

Larger multi-centre studies that particularly focus on confirming the efficacy of mobile phone technology and identifying the cost-effectiveness of the strategy in improving adherence among the ART population in Malaysia are hereby recommended.

## Supporting information

S1 File Consort 2010 checklist. (PDF)

S2 File Trial Protocol. (PDF)

S3 File Related manuscript. (PDF)

## Abbreviations

Adhwk1: Adherence ratio yesterday*
Adhwk2: Adherence ratio 2 days ago*
Adhwk3: Adherence ratio 3 days ago*
Adhwk4: Adherence ratio 4 days ago*
*Adherence ratio: [1 –(number of doses missed/number of doses prescribed)]
Followsch: How closely drug schedule was followed over the past 4 days
Instfu: How closely special instructions with medications were followed over the past 4 days
Lastskip: When last any medication was skipped
