# Supplementary material for: Mobile phone reminders and peer counseling improve adherence and treatment outcomes of patients on ART in Malaysia: A randomized clinical trial
Source: PLoS One. 2017 May 16;12(5):e0177698. doi: 10.1371/journal.pone.0177698 (PMC5433794; doi:10.1371/journal.pone.0177698)
Supplement: S2 File — (PDF) [file pone.0177698.s002.pdf]

EFFECTIVENESS OF MOBILE PHONE TECHNOLOGY IN IMPROVING ADHERENCE AND CLINICAL OUTCOMES AMONG HIV POSITIVE PATIENTS ON ANTIRETROVIRAL THERAPY – A RANDOMIZED CONTROLLED TRIAL

DR. SURAJUDEEN ABIOLA ABDULRAHMAN

PROF. (DR) LEKHRAJ A/L GYANCHAND RAMPAL

ASSOC. PROF. DATO' DR. FAISAL HJ IBRAHIM

DR. HAYATI KADIR@SHAHAR

DR. ANURADHA P. RADHAKRISHNAN

JUNE 2013

A Research Proposal submitted to the Department of Community Health, Faculty of Medicine and Health Sciences, Universiti Putra Malaysia

INTRODUCTION

A. Background

HIV was first diagnosed in Malaysia in 1986 (Malaysia NSP, 2011-2015). By end of 2011, 94, 841 HIV cases, 17,686 AIDS cases and 14, 986 deaths had been cumulatively reported to MOH (Malaysia 2012 Global AIDS Response Country Progress Report). As at 2011, 81,000 persons were living with HIV and 3,479 new infections in the same year (UNAIDS Global Report, 2012). Current National Prevalence is 0.5% (Malaysia NSP, 2011-2015). The epidemic is concentrated among IDUs, MSM, FSWs and Transgender people but recently an increasing heterosexual spread has been observed (Malaysia NSP, 2011-2015). Males are generally more affected (90%) but there's an increasing trend in females (21% in 2011) (Malaysia NSP, 2011-2015). About 14,000 HIV infected are currently on free first-line ARVs and heavily subsidized second-line treatment from Government-funded ART program (Malaysia 2012 Global AIDS Response Country Progress Report). However, 50-60% unmet need for ARVs still exist (UNAIDS, 2011).

The goal of HIV treatment is to reduce morbidity, prolong survival, improve quality of life, improve immunologic functions and sustain viral suppression (Lee Preininger et al, 2011). Medication adherence is the cornerstone of long-term HIV suppression (Lee Preininger et al, 2011). Adherence to ARVs prevents disease progression and emergence of resistance mutations. It also reduces morbidity and need for frequent, complicated regimen (Lee Preininger et al, 2011). Minimum adherence levels of 95% are required for treatment success (WHO, 2005). Barriers to adherence include stigma & discrimination, pill burden, disclosure issues, depression, unemployment, low family income. Other barriers include lack of community support & integration, ineffective adherence counseling (before and during treatment). Innovative ways of overcoming these barriers are urgently required. WHO classifies HIV/AIDS into 4 stages and success of ART programs is measured by patient retention (Penny Leithwaite, Ed Wilkins, 2009).

Problem statement: Poor adherence is a major threat to successful ART outcome in Malaysia (Malaysia NSP, 2011-2015) and generates major concerns about growing resistance of HIV to the currently available ARVs (WHO, 2007). Poor adherence leads to treatment failure, disease progression and death (WHO, 2007). With treatment failure, the preventive opportunity that ARVs provide is also lost (WHO, 2007). Poor adherence has grave socioeconomic impact on program funding – second line medications are more expensive and complex (WHO, 2007). Effectiveness of routine adherence counseling on treatment adherence among ART patients has not been previously studied in Malaysia. Similarly, factors affecting adherence to ART has not been extensively studied in Malaysia.

Significance of research: This research is in line with Key activity 6 of Strategy 2 in the current drive by Malaysian Government to improve adherence to treatment and detection of treatment failure (Malaysia NSP, 2011-2015). It is also consistent with current WHO recommendation of interventions to optimize adherence to ART (WHO, 2013). It will contribute to existing body of knowledge on factors associated with treatment default and LTFU among HIV patients on ART. Its results will provide baseline information on effectiveness of current standard of care (routine adherence counseling) on treatment adherence and clinical outcomes. It will provide an opportunity to test the effectiveness of the intervention as a tool/strategy to improve patient retention. The outcome will provide information to Government for policy decisions in improving service delivery for HIV patients, reduce cost of HIV treatment programs and scale-up to other locations across Malaysia.

Objectives of study:
The general objective of this study is:

- To determine the effectiveness of mobile phone technology (SMS and Phone call reminders) in improving clinic attendance, medication adherence and outcome of treatment among HIV patients on ART.

The Specific objectives include:
1. To determine socio-demographic factors that affect treatment adherence and contribute to treatment default and Lost-to-follow-up (LTFU) among HIV patients on ART.
2. To develop and implement a 'reminder module' (delivered via SMS and telephone calls) to improve clinic attendance and medication adherence among HIV patients on ART.
3. To determine the effectiveness of SMS and telephone call reminders in improving clinic attendance and medication adherence among HIV patients on ART.
4. To determine the effect of medication adherence on the clinical and immunological outcome of HIV patients on ART.

Research hypotheses:

H_1: There is no significant difference in the socio-demographic characteristics of HIV patients who adhere very well to their ART compared to those who do not.

H_2: The application of mobile phone technology (SMS and phone call reminders) will significantly improve treatment adherence and clinical outcomes of HIV patients on ART, when compared to routine adherence counselling and paper-based appointment scheduling alone.

H_3: There is a strong and direct relationship between Good adherence to treatment (regular clinic visits and strict medication adherence) and outcome of antiretroviral treatment among HIV patients on ART.

LITERATURE REVIEW

Epidemiology of HIV/AIDS: AIDS was first recognized in 1981 to be caused by HIV-1. HIV-2 causes a similar illness but is less aggressive and restricted mainly to West Africa. Continuous high level of replication of the virus leads to suppression of the immune system through immune-mediated destruction of the CD4 lymphocytes and increased morbidity as patients become more susceptible to other types of opportunistic infections (Penny Lewthwaite, Ed Wilkins, 2009). HIV-1 can be further sub-divided into three groups (M, O and N) and genetic subtypes. Nine subtypes are currently recognized for group M (A-K), with numerous sub-subtypes (e.g A1-A4) and circulating recombinant forms (e.g CRF01_AE). Each of these tends to be associated with a particular geographic area with less strong association for transmission categories, rate of progression and resistance patterns. Sub-type B is most prevalent in Americas and Europe, but globally sub-types C is known to account for half of all strains. HIV-2 infection differs from HIV 1 in being inherently resistant to NRTIs, and patients have lower viral loads, slower CD4 decline, lower rates of vertical transmission and 12-fold slower progression to AIDS (Penny Lewthwaite, Ed Wilkins, 2009). Though the exact mechanism underlying the CD 4 decline in HIV is yet to be fully understood, it is known to correlate inversely with the plasma viral load, but is not restricted to virus-infected cells. Both are monitored closely in patients and used as measures of disease progression. Virus-specific CD8 cytotoxic T lymphocyte cells develop rapidly after infection and are the most important elements in recognizing and lysing infected CD4 cells. They play a crucial role in controlling HIV replication after infection and in the subsequent rate of disease progression (Penny Lewthwaite, Ed Wilkins, 2009).

Modes of transmission: HIV is present in blood, semen, and other body fluids such as breast milk and can be transmitted through heterosexual route (>75%) and from mother to child (5-10%) with wide racial and ethnic variations among countries' subpopulations as a result of immigrants influx from high prevalence countries. The incidence in injecting drug users also vary from country to country with UK having <1% and >50% in Eastern Europe, Vietnam, India, China and Malaysia.

Clinical history and features: Primary infection is characterized by sero-conversion illness in 70-80% of patients owing to high levels of circulating HIV 1. Common symptoms range from fever (80%), malaise (68%), arthralgia (54%), macula-papular rash (51%), myalgia, oral ulcers and pharygitis: neurological complications such as GBS and Bell's palsy occasionally occur. Most symptoms generally resolve in 7-10 days though the illness is more severe in a few patients as a result of opportunistic infections (eg PCP, esophageal candidiasis) reflecting a more profound CD4 cells depletion often to as low as <200cells/mm3 (Penny Lewthwaite, Ed Wilkins, 2009). Patients with severe symptoms are more likely to have a rapid disease progression, though the illness is mild in many patients and only recognized through retrospective enquiry. Symptomatic recovery parallels a rise in CD4 count and viral load suppression. Diagnosis is made by detection of HIV RNA in serum or immunoblot assays (antibody detection). The appearance of specific anti-HIV antibodies in serum (sero-conversion) takes place later at 2-12 weeks (median 8weeks) (Penny Lewthwaite, Ed Wilkins, 2009). In asymptomatic infection, the CD4 count increases again usually below its pre-infection level and viral load stabilizes at a particular set point for several years. Patients with CD4 count of 350-800 cells/mm3 are usually well. The period between infection and development of symptomatic or late-stage disease varies. Infected individuals may be completely asymptomatic or have generalized lymphadenopathy (CDC classification category disease A, WHO stage 1).
Without treatment, the CD4 count eventually declines and at CD4 count <350, the individual becomes increasingly susceptible to a wide range of opportunistic infections ranging from TB, Pneumonia, Herpes zoster, recurrent oral and vaginal candidiasis and oral hairy leukoplakia. This is observed at CD4 count 200-350 cells/mm3 and is typical of CDC classification category disease B or WHO stage 2 & 3.
At CD4 count <200 cells/mm3 other opportunistic infection or HIV-related tumor may develop. AIDS (CDC classification category disease C or WHO stage 4) is defined by the appearance of other opportunistic infections and tumors such as PCP, cerebral toxoplasmosis, Kaposi's sarcoma, oesophageal candidiasis and diarrhoea diseases.

With CD4 count <100cells/mm3, disseminated Mycobacterium avium intracellulare infection, systemic fungal infections, cytomegalovirus infections, neurological problems such as PML and Non-Hodgins Lymphomas may occur.

Immune response to HIV: Without Antiretroviral therapy, the median time to a CD4 count of 200cells/mm3 which is the critical thresh hold for opportunistic infections, is 8-10 years (Andrew McMichael, Lucy Dorrell, 2009). Poor medication adherence to ART and a host of other immunologic mechanisms such as early establishment of a reservoir of latently infected CD4 cells, CD8 dysfunction, immune dys-regulation and a host of other mechanisms contribute to failed immune control in HIV infection.

Antiretroviral Therapy: Antiretroviral therapy uses agents in combination that act at different stages of the virus life-cycle. Currently known ARVs fall into 6 different categories, namely: (a) Nucleoside analogue reverse transcriptase inhibitors (NRTIs) such as Zidovudine, Lamivudine, Didanosine, Emtricitabine etc (b) Nucleotide analogue reverse transcriptase inhibitors eg Tenofovir (c) Non-nucleoside reverse transcriptase inhibitors (NNRTIs) such as Nevirapine, Efavirenz and Etravirine (d) Protease Inhibitors such as Ritonavir, Indinavir, Saquinavir etc (e) Entry Inhibitors such as Enfuviritide and Maraviroc (f) Integrase inhibitors eg Raltigravir (Alasdair Breckenridge, 2009). These drugs are often given in combinations in order to achieve maximum viral suppression and prevent resistance to any one of the drugs by the virus. These classes of drugs are characterized by unique pharmacodynamic and pharmacokinetic properties, adverse effects, drug interactions and cross resistance between and among groups.

Adherence to treatment: According to the WHO Adherence Project, 2003, adherence can be defined as "the extent to which a person's behaviour – taking medications, following a diet, and/or executing lifestyle changes, corresponds with the agreed recommendations from a health care provider". Medication adherence may also be defined as the extent to which a patient takes his or her medications in the way intended by the health care provider (Machtinger, E.L., Bangsberg, D.R., 2005). Medication adherence in HIV/AIDS has been defined by Jani (2004:1) as "the ability of a person living with HIV/AIDS to be involved in choosing, starting, managing and maintaining a given therapeutic combination medication regimen to control viral replication and improve immune function". According to WHO (2005), minimum adherence levels of 95% are required for treatment success (Fairly et al., 2005; Jani, 2004; Paterson et al., 2000; Saple, 2005). Other studies have demonstrated that ARV medication adherence levels of 54 – 95% is required to maintain prolonged viral load suppression depending on the allowable flexibility margins of each ART program. It is however generally accepted that those clients who adhere strictly to their medication achieve viral suppression, while those who are not adherent may not (Lee Preininger et al., 2011). Adherence is a primary determinant of the effectiveness of treatment because poor adherence attenuates optimum clinical benefit. Good adherence improves the effectiveness of interventions, aimed at promoting healthy lifestyles such as diet modification, improved physical activity, non-smoking and safe sexual behaviours, and of the pharmacological-based risk-reduction interventions. It also affects secondary prevention and disease treatment interventions (WHO, 2003). Treatment adherence also involves regular clinic attendance in line with schedules jointly determined by the patient and the health care provider, based on approved international protocols for standard care of a particular disease. The success of antiretroviral treatment has been widely correlated with strict medication adherence (adherence >95%), behavioural and lifestyle modifications, disclosure issues, nutrition, psychosocial support, issues of stigma and discrimination, employment status and family income, in different studies. However, most studies have identified strict medication adherence as the single most important predictor of immunologic, viral and clinical outcome of treatment in HIV clients. Adherence measurements can be grouped into measures based on a patients self-report of pill-taking behaviour and measures that are objective surrogates of pill-taking behaviour such as pill count or MEMS cap (Machtinger and Bangsberg, 2005).

Factors associated with non-adherence: In general, factors associated with non-adherence include pill burden, frequency of drug dosing, dietary requirements, depression, drug and alcohol abuse, medication side effects, patient-provider relationship, co-morbidities, among others. Some studies have also shown low income and education to be predictors of non-adherence while socio-demographic factors are only surrogate factors for predicting adherence (Joan M. Duggan et al., 2009; Talam N.C. et al., 2008; Allen C.F. et al., 2011).

Interventions to improve adherence: Interventions to improve adherence encompass: (1) Patient Education and Collaborative Planning (2) Adherence Case Management (3) Directly Observed Therapy (4) Simplified Treatment Regimens and (5) Adherence Devices – including medication organizers (pill boxes), reminder devices/strategies, and visual medication schedules (Machtinger and Bangsberg, 2005). A number of recent studies have shown the effectiveness of mobile phone technology (Short Text Messages and telephone call reminders) in improving medication adherence and clinical outcomes among HIV patients on ART (Hovart T. et al., 2012; Lester R.T. et al., 2010; Pop-Eleches C. et al., 2011; M.H.M.M.T van Velthoven et al., 2013; Reynolds N.R.et al., 2008; Free C. et al., 2013). No such/similar study has been conducted in Malaysia.

RESEARCH APPROACH

B. Research methodology

Study Location: The study will be conducted in Hospital Sungai Buloh, Selangor, Malaysia.

Study Design: It will be an experimental (randomized Controlled Trial) study design, single-blinded among newly diagnosed HIV positive patients who are eligible to commence ART from December 2013 to August 2014. Following enrollment and allocation to study arm, baseline adherence, clinical symptoms, CD4 count, viral load, Weight, TB status and Opportunistic infection index will be assessed and measured for the 2 groups, prior to the commencement of ART. The intervention group will receive appointment and medication reminders by text-messaging and telephone calls, in addition to standard care. Based on the Theory of Planned Behaviour Model (Azjen, 1985), appointment reminders will be sent by Short Text Message (in Malay and English languages) three days prior to each individual client's scheduled appointment and telephone call reminders a day prior to the scheduled clinic appointment visits. Weekly short text messages will also be sent to clients to remind them of taking their medications. The control group will only receive standard care and paper-based appointment during clinic visits. Both groups of clients will be followed up for a period of 6 months on ART. Repeated CD4 count, viral load, Weight, TB status and OI index assessment and measurements will be conducted at 3 and 6 months, respectively to determine treatment outcome. Medication adherence will be measured using structured self-report questionnaires. Clinic visits will be measured through medical records of clinic attendance and clinicians' notes.

Study Duration: Recruitment and data collection will be from December 2013 to February 2015.

Study population: The study population is all HIV positive clients assessed and found to be eligible for ART commencement at Hospital Sungai Buloh, Selangor

Inclusion criteria: Age range of 15 to 65 years eligible to commence ART, able to read text messages and have a mobile phone access.

Exclusion criteria: Clients who are previously or currently on ART, foreigners & pregnant patients

Sampling frame: It is the list of all HIV positive clients assessed and found to be eligible for ART from December 2013 to August 2014 in Hospital Sungai Buloh, Selangor

Sampling unit: Individual patients who are HIV positive but ARV-naive, assessed and found to be eligible to commence ART based on CD4 < 500 cells/ml and/or WHO stage 3 & 4 disease.

Sampling method: A simple random sampling method will be used to select eligible patients for the study.

Random allocation: Participants will be allocated to either of the intervention or control groups based on simple, complete randomization technique using their unique ID numbers from a random number table. An independent project biostatistician will generate 1:1 randomization numbers for study arm assignments using a random number generating program. Random allocation will consider and eliminate all forms of possible bias based on the socio-demographic characteristics of the clients.

Allocation concealment: Written allocation of assignment will be sealed in individual opaque envelopes marked with study identification numbers which will be available in the study clinic to allocate the target number of participants. After consenting to participate and meeting the inclusion criteria, screened subjects will be enrolled and immediately afterwards are assigned to a randomized study arm by the study coordinator opening the sealed envelopes to determine allocation. Age and gender will be assessed for balance of study arm allocation.

Blinding: Investigators will be blinded to intervention assignments.

Sample size: The formula for hypothesis testing was used (Lameshow et al., 1990).

$n = 2d^2[Z1\text{-}a/2 + Z1\text{-}\beta]^2\ \mu1\text{-}\ \mu2$

Where: d = estimated standard deviation (assumed to be equal for each group) is 132 cells/µl (Ref: 10, Table 1)

µ1= estimated mean CD4 count increase after commencement of ART with >90% adherence is at least 50cells/µl (14.3%) i.e 550±50

µ2= estimated mean baseline CD4 count at commencement of ART (Based on WHO 2013 guideline of when to commence ART, which is CD4 = 500 cells/ml of blood)

Z1-a/2 = standard error when a = 0.05 (95% Confidence Interval) = 1.96

Z1-ß = standard error associated with power = 0.842 (ß = 0.20) Power (1-ß) = 80%

Expected CD4 increase within 6months of treatment with >95% adherence is 50 cells/ml

n = 2 x $(132)^2[1.96+0.842]^2/(550-500)$

n = 110 which is the minimum required sample size 110 + 10% of 110= 121 (attrition)

121 x 2 = 242 (for experimental and control groups)

Hence, sample size of 121 per group will be used for the study

Intervention Protocol: For the intervention group – a 'reminder module' will be developed and delivered via SMS and telephone calls by 2 PLHIV (research assistants). This will include weekly SMS medication reminders, SMS reminders 3 days prior to scheduled clinic appointments and telephone call reminders a day prior to scheduled clinic appointment (in addition to standard care – adherence counseling). To ensure confidentiality, typical medication reminder text messages will include a short slogan in Malay language "Apa khabar" "Ini untuk menberithau anda ubat" meaning "How are you?" "This is to remind you of your medications". Appointment reminder text message would be "Apa khabar" "Tolong ingat tarikh temu janji lusa" meaning "How are you?" "Remember your appointment day after tomorrow" and telephone conversation would be standardized and short, with the message "Apa khabar" "Tolong ingat tarikh temu janji besok" meaning "How are you?" "Remember your appointment tomorrow". The control group will receive standard care - routine adherence counseling only. Patients will not be required to provide any responses to the text messages. A call and SMS log will be recorded and kept. Two PLHIV that are stable and have been consistent and adherent to ARV medications for at least 2 years will be recruited as research assistants. Using standard training module developed and validated by a team of experts from Universiti Putra Malaysia and Hospital Sungai Buloh, a two-day onsite training of the research assistants on HIV prevention methods, care and treatment issues and how to respond to common questions related to medication adherence, including medication side effects, when asked by study participants, will be conducted. In addition to sending SMS and making telephone calls to patients, they will also be required to provide counseling support and provide guidance to patients (when required) in filling out medication adherence questionnaires during every clinic visits. Patients in the intervention group will be further divided into 2 sub-groups, each under the supervision of one research assistant, who will be responsible for providing them with counseling and psychosocial support, in addition to the care and management provided by the clinicians and nurses, throughout the duration of the study. Each patient in the intervention group would undergo a minimum of three (during clinic visits at month 1, month 3 and month 6) individual counselling sessions with the research assistants lasting an average of 15 minutes per encounter. An intervention manual containing the training modules and implementation guidelines will be provided to the research assistants as spiral-bound hard copy reference document, to guide their interactions with the patient and assure quality and uniformity in the implementation of the intervention.

Data collection: Research data will be collected using the below plan:

- ***How***: 2 types (Primary and Secondary) of data will be collected from 3 main sources;

(a) Hospital medical records – Patients' socio-demographic and clinic attendance data will be collected from hospital records; pharmacy records will be utilized for measuring adherence to drug refill appointments, while clinicians' notes will be assessed for clinical information regarding weight, TB status, and OI index of clients (through standardized OI monitoring forms). Laboratory records will be accessed for baseline and follow-up CD4 count and viral load test results.

(b) Structured questionnaire and standardized medication adherence monitoring forms would be used to collect information from clients' medication adherence self-reports during interaction with clinician/nurses/pharmacists at every scheduled clinic visit/appointment.

- ***Who and When***: Socio-demographic (at enrolment) and clinic visit information (at every visit) will be collected by clinic medical record officers, laboratory information by laboratory scientists (at baseline, Months 3 and 6) and clinical and medication adherence information will be collected by clinicians/nurses/pharmacists at every client visit.

A log book on phone calls and text messages will also be recorded.

- ***Duration***: Data will be collected over a 13 months period.
- ***Questionnaire***: The Adult AIDS Clinical Trial Group (AACTG) adherence questionnaires (Chesney, M.A. et al, 2000) will be adapted with slight modifications, for the purpose of this study. Specialized questionnaires will be used at baseline and follow up. The questionnaire will be pre-tested on a population not included in the sample for validity and reliability.

The baseline questionnaire shall consist of sections A – I (A: Patient's readiness and understanding of treatment plan, B: Patient's social support and disclosure status, C: Possible reasons for missing medications, D: Assessment of time and duration of missed doses, E: Assessment of psychosocial stress/difficulties in the past one week, F: Assessment of psychosocial stress/difficulties in the past one month, G: Assessment of alcohol and drug use profile (past & current, degree of usage), H: Patient's background (socioeconomic characteristics, sexual orientation and behaviour) and I: Symptom review of the past 4 weeks). Responses to questions A-C, E will be graded on a 4-point scale, question D, F & I on a 5-point scale, G on a 6-point scale, H will have 7 questions on socioeconomic and demographic characteristics.

The follow questionnaire shall consist of sections A – H (A: Review of current medication and number of missed doses and days of missed doses, B: Number of doses missed in the past 4 days, C: How closely the patient followed dosing schedule, D: Review of any dietary specifications with the drugs, E: Whether the patient missed medications on weekends, F: When was the last time the patient missed medications, G: Possible reasons for missing medications, H: Symptom review of the past 4 weeks). Responses to questions C, D, F & H are graded on a 5-point scale, E on a 2-point scale, and G on a 4-point scale.

Type of information	Source	Responsible person	Time of collection
Socio-demographic	Medical records	Medical records officer	At enrolment
Clinic visits	Medical records	Medical records officer	Every visit
Medication refill	Pharmacy records	Pharmacists	Every visit
Clinical (Wt, TB & OI status)	Clinician's notes	Clinicians/nurses/adherence counsellors	Every visit
Immunological (CD4)<br>Virological (viral load)	Laboratory records	Laboratory scientists	Baseline & 6mths
Medication adherence	Adherence questionnaire	Research assistants/nurses/adherence counsellors	Baseline, then every visit (particularly 3&6mths)

- ***Data Storage***: All data generated from this study will be stored for a maximum of 3 years after the completion of the study, after which they will be destroyed. However, during this period they will remain joint intellectual property of Universiti Putra Malaysia and Hospital Sungai Buloh.

Primary Outcomes: The first primary outcome is (improved) patient adherence to ART at six months. This is assessed by self-report using a standardized questionnaire of the number of pills missed in the last 30 days and calculating percent adherence based on the expected number of doses taken and/or number of missed doses. Percent adherence will be analyzed as continuous variable and polytomous variable. Subjects will be considered as "Good adherence" when they achieve an optimal adherence cut of >95% of medications taken as directed with <3 missed doses in the last 30 days period. "Fair adherence" will be considered as 80-95% of medications taken as directed with 3-8 missed doses in the last 30 days period, while "Poor adherence" will be considered as <80% of medications taken as directed with >8 missed doses in the last 30 days period. Medication adherence will be assessed at each follow-up visit, particularly at months 3 and 6. The six month scheduled follow-up is the primary analysis but the three month assessment will allow for evaluating trends but is not the primary outcome.

The second primary outcome is (regularity of) scheduled clinic attendance at six months. This will be assessed from records of scheduled clinic visits in the medical records unit and supported by record of scheduled drug pick-up in the pharmacy. Regularity of scheduled clinic visits will be calculated based on number of scheduled clinic visits attended and/or number of times defaulted. Number of missed appointments will be analyzed as a continuous variable and polytomous variable. Subjects will be considered as "Regular clinic attendee" if they never missed any scheduled clinic appointment. A "Defaulter" will be a patient who has missed one or more scheduled clinic appointment for any reason(s), while a patient will be considered "Lost-to-follow-up" if s/he refuses to show up for scheduled clinic visit for 3 consecutive months, after 3 consecutive attempts to track the client and bring them back on treatment. The six month scheduled follow-up is the primary analysis but the three month assessment will allow for evaluating trends.

Secondary Outcomes: The first secondary outcome is (improved) clinical status of patients at six months. This will be assessed by measuring their weights, assessing their TB and opportunistic infection status at every clinic visits, particularly at three and six months and comparing these with the baseline status. Patients' weight is routinely measured and recorded by nurses at the study site, as part of standard patient management protocol. These records will be accessed and utilized in the analysis. Patients' TB status is also routinely assessed and recorded by clinicians/nurses/adherence counselors at every clinic visits. These records will be accessed and utilized to categorize patients into four groups, namely "No signs and symptoms of TB", "TB suspected and referred for evaluation", "Currently on INH prophylaxis" and "Currently on TB treatment". Opportunistic infection status will be assessed through WHO clinical staging at every clinic visits. The six month scheduled follow-up is the primary analysis but trend will be evaluated at three months.

The other secondary outcome is (improved) immunologic status/response of patients at six months. This will be assessed based on records of two (2) laboratory measurements – CD4 T cell count and viral load. Patients' CD4 count results at six months will be assessed and compared with baseline values to determine if there's a consistent increase (or otherwise) as expected with strict medication adherence. If patients are adherent to ART regimens, absorb the drugs normally, and if their virus is not resistant to the ART drugs in the regimen, it is expected that their CD4 count should increase from baseline levels by at least 50 cells per microliter of blood within 6-12 months of initiating treatment. CD4 count value at six month scheduled follow-up is the primary analysis.

Similarly, plasma HIV RNA load results at six months will be assessed and compared with baseline levels to determine if there is a significant suppression at six months. If patients are adherent to ART regimens, absorb the drugs normally, and if their virus is not resistant to the ART drugs in the regimen, it is expected that their plasma HIV load should be suppressed to undetectable levels (<400 copies/ml) by six months and remain suppressed thereafter. Analysis of "suppressed" (<400 copies/ml) versus "failure to suppress" (>400 copies/ml) at six months as a dichotomous variable is the primary analysis.

Data analysis: Data collected will be collated, checked, cleaned, entered into and analyzed using Statistical Package for Social Sciences software (SPSS) version 21 and AMOS software. Parametric tests (T-test, repeated measures ANOVA) and non-parametric tests will be conducted on the data. P value for test of significance of results will be set at 0.05, alpha level (Type 1 error) at 0.05 (Confidence Interval of 95%), Power at 80% to detect an impact of 15% in the fraction of patients with adherence of at least 90% (assuming a no intervention median adherence level of 90%) using a two-tailed test, Z=1.96, and strength by effect size analysis. Analysis will be by intention-to-treat and complete case analysis.

Independent variable	Dependent variable	Type of analysis
**Categorical** (socio-demographic, behavioural & adherence factors)	**Continuous** (Wt, CD4, viral load)	**T-test, Repeated Measures ANOVA**
**Categorical & Continuous (All IVs)**	**Categorical** (adherence level, TB status, OI index, viral suppression)	**Multivariate/Multinomial Logistic Regression Model**
**Categorical** (All IVs converted to categorical)	**Categorical** (All DVs converted to categorical)	**-Structural Equation Modelling**<br>**-Chi-square (sub-group analysis)**

Limitations: (1) Self-report of adherence – exaggeration of true adherence (Pharmacy refill records, CD4 count and viral load will provide a means of validating the responses)
(2) Pill count will not be used
(3) Structured questionnaires may not elicit in-depth information

Ethical considerations: The study and materials will be presented to the UPM Ethics approval committee and the Malaysian Ministry of Health's Institutional Review and Ethics Committee for review and approval. Thereafter, permission will be sought from the Medical Director of the hospital in writing to use their clients. The researcher will work in collaboration with attending clinicians, nurses and pharmacists in identification and monitoring of the clients.

Expected outcomes: The expected outcomes of this research include:

1. A significant difference in treatment adherence (medication adherence and regular, scheduled clinic attendance) is expected in the intervention group compared to the control group.
2. Better clinical outcome in the intervention group compared to the control group.
3. Socio-demographic, psychosocial and other risk factors that determine differences in treatment adherence between the intervention and control groups will be identified.
4. Provide baseline information on the effectiveness of current standard of care (routine adherence counselling) on treatment adherence and clinical outcomes.

Ghantt chart:

**Year**	**2013**												**2014**												**2015**	
**Project (Activities)**	**J**	**F**	**M**	**A**	**M**	**J**	**J**	**A**	**S**	**O**	**N**	**D**	**J**	**F**	**M**	**A**	**M**	**J**	**J**	**A**	**S**	**O**	**N**	**D**	**J**	**F**
Proposal writing			■	■	■																					
Ethical approval							■	■																		
Proposal presentation						■	■																			
Validation of questionnaire									■	■																
Data collection											■	■	■	■	■	■	■	■	■	■	■	■	■			
Interim Data Analysis															■	■										
Final Data Analysis																								■	■	
Report Writing																								■	■	■
Compilation/writing article																									■	■

Budget: Total cost of the research is estimated at 110,000 RM (See details in appendix)

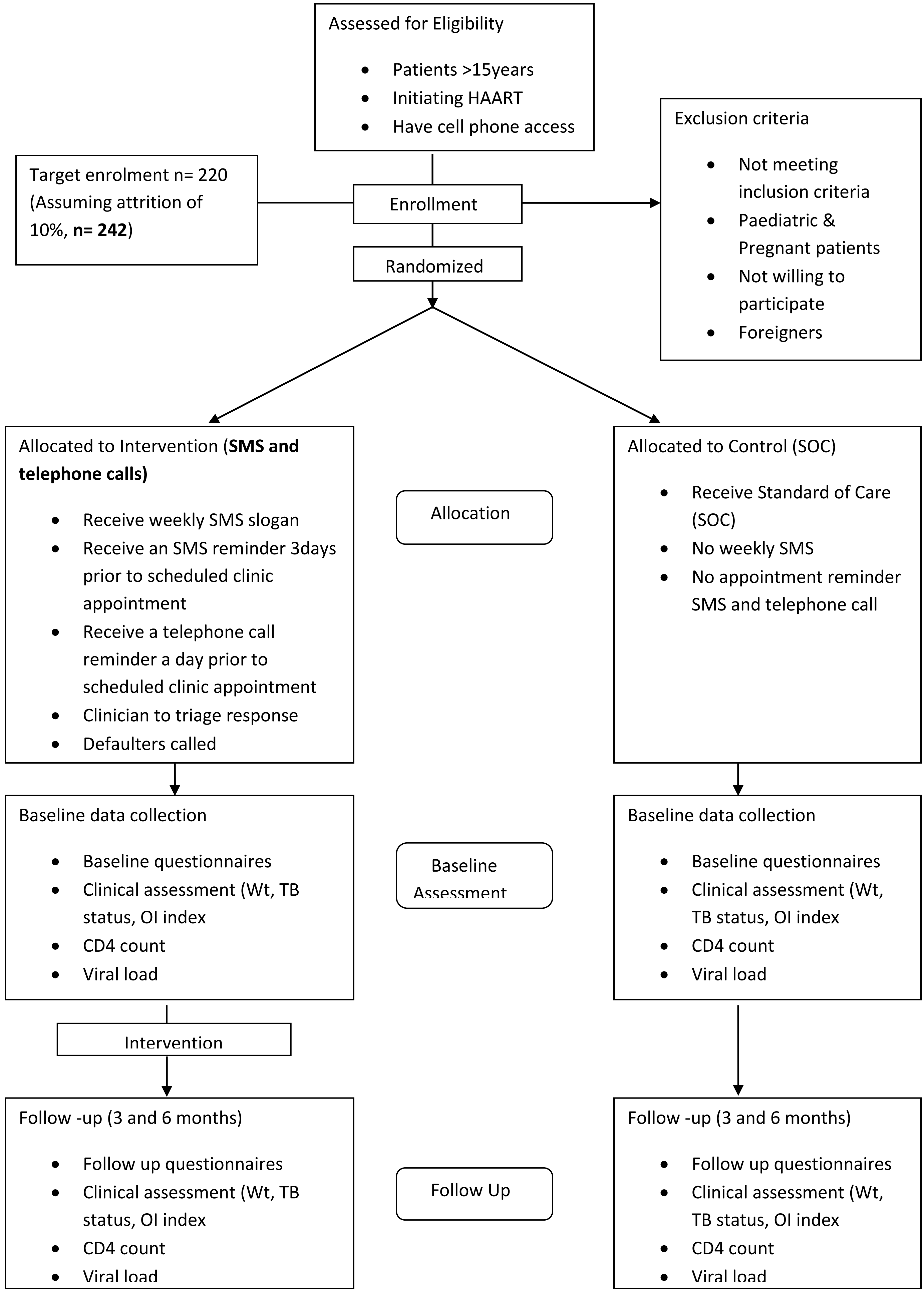
Assessed for Eligibility
Patients >15years
Initiating HAART
Have cell phone access
Exclusion criteria
Not meeting inclusion criteria
Paediatric & Pregnant patients
Not willing to participate
Foreigners
Target enrolment n= 220 (Assuming attrition of 10%, **n= 242**)
Enrollment
Randomized
Allocated to Intervention (**SMS and telephone calls)**
Receive weekly SMS slogan
Receive an SMS reminder 3days prior to scheduled clinic appointment
Receive a telephone call reminder a day prior to scheduled clinic appointment
Clinician to triage response
Defaulters called
Allocation
Allocated to Control (SOC)
Receive Standard of Care (SOC)
No weekly SMS
No appointment reminder SMS and telephone call
Baseline data collection
Baseline questionnaires
Clinical assessment (Wt, TB status, OI index
CD4 count
Viral load
Baseline Assessment
Baseline data collection
Baseline questionnaires
Clinical assessment (Wt, TB status, OI index
CD4 count
Viral load
Intervention
Follow -up (3 and 6 months)
Follow up questionnaires
Clinical assessment (Wt, TB status, OI index
CD4 count
Viral load
Follow Up
Follow -up (3 and 6 months)
Follow up questionnaires
Clinical assessment (Wt, TB status, OI index
CD4 count
Viral load

References:

1. United Nations/WHO (2003) 'Workshop on HIV/AIDS and Adult Mortality in Developing Countries' New York, 8-13 September 2003
2. United Nations (2011) 'Political Declaration of HIV/AIDS: Intensifying our Efforts to Eliminate HIV/AIDS'
3. UNAIDS (2011, November) 'World AIDS Day Report 2011'
4. UNAIDS (2011, November) 'World AIDS Day Report 2011'
5. UNAIDS (2010) 'UNAIDS report on the global AIDS epidemic'
6. UNAIDS (2011, November) 'World AIDS Day Report 2011'
7. Malaysia National Strategic Plan 2011 – 2015
8. Lee Preininger et al (2011): Long term Medication adherence in patients receiving Antiretroviral therapy, Current HIV Research, 2011, 9, 253-255
9. Hovart T, Azman H, Kennedy GE, Rutherford GW (2012): Mobile phone text messaging for promoting adherence to antiretroviral therapy in patients with HIV infection; Cochrane Database of Systematic Reviews, 2012, Issue 3, Art. No.: CD009756, DOI: 10.1002/14651858.CD009756
10. Lester RT, Ritvo P, Mills EJ, Kariri A, Karanja S, Chung MH, et al. (2010): Effects of a mobile phone short message service on antiretroviral treatment adherence in Kenya (WelTel Kenya 1): a randomized trial. Lancet 2010, Nov. 27; 376 (9755): 1838-45
11. Pop-Eleches C, Thirumurthy H, Habyarimana JP, Zivin JG, Goldstein MP, de Walque D, et al. (2011): Mobile phone technologies improve adherence to antiretroviral treatment in a resource-limited setting: a randomized controlled trial of text message reminders. AIDS 2011; 26(6): 825-34
12. M.H.M.M.T van Velthoven et al (2013): Scope and effectiveness phone messaging for HIV/AIDS care: A systematic review, Psychology, Health & Medicine, 18:2, 182-202
13. WHO (2007) 'Prioritizing Second-Line Anti-retroviral Drugs for Adults and Adolescents: a Public Health Approach'. Report of a WHO Working Group Meeting, 21-22 May, 2007
14. WHO (2013) 'Consolidated guidelines on the use of antiretroviral drugs for treating and preventing HIV infection, recommendations for a public health approach'
15. Penny Lewthwaite, Ed Wilkins (2009): Natural History of HIV/AIDS, Medicine, Vol. 37, Issue 7, 333-337
16. Andrew McMichael, Lucy Dorrell (2009): The Immune response to HIV, Medicine, Vol. 37, Issue 7, 321-325
17. Alasdair Breckenridge (2009): Pharmacology of Drugs for HIV, Medicine, Vol. 37, Issue 7, 374-377
18. Machtinger, E.L., Bangsberg, D.R.,(2005): Adherence to HIV Antiretroviral Therapy. Available from http://hivinsite.ucsf.edu/Insite?page=kb-03-02-09#S1X (accessed 09/03/13)
19. WHO (2003): Adherence to long-term therapies: evidence for action
20. Joan M. Duggan, Ann Locher, Brian Fink, Chrystal Okonta and Joana Chakraborty (2009): Adherence to antiretroviral therapy: A survey of factors associated with medication usage, AIDS Care, Vol. 21, No. 9, September 2009, 1141-1147
21. N.C Talam, P. Gatongi, J. Rotich, S. Kimaiyo (2008): Factors affecting antiretroviral drug resistance among HIV/AIDS adult patients attending HIV/AIDS clinic at Moi Teaching and Referral Hospital, Eldoret, Kenya, East African Journal of Public Health, Vol. 5, No. 2, 74-78
22. CF Allen, Y. Simon, J. Edwards, DT Simeon (2011): Adherence to antiretroviral therapy by people accessing services from Non-governmental HIV support organizations in Three Caribbean countries, West Indian Med. Journal, Vol. 60, Issue 3: 269-275
23. Fairly C.K, Permana, A., Read, T.R.H., (2005): Long term utility of measuring adherence by self report compared with pharmacy record in a routine clinic setting, HIV Medicine 6, 366-369
24. Jani A.A., (2004): Adherence to HIV treatment regimens: recommendations for best practices, pp. 3-72. Available from http://www.alpha.org/ppp/hiv
25. Paterson, D.L., Swindells, S., Mohr, J., Brester, M., Vergis, E. N., Squire, C., Wagener, M.M., Singh, N., (2000): Adherence to protease inhibitor therapy and outcomes of patients with HIV infection, Annals of Internal Medicine 133 (1), 21-30
26. Nancy R. Reynolds, Marcia A. Testa, Max Su, et al., (2008): Telephone Support to Improve Antiretroviral Medication Adherence – A Multisite Randomized Controlled Trial. Journal of Acquired Immune Deficiency Syndrome, Vol. 47, No. 1, January 1, 2008
27. Free C, Phillips G, Galli L, Watson L, Felix L, et al. (2013): The effectiveness of Mobile-Health Technology-Based Health Behaviour Change or Disease Management Interventions for Health Care Consumers: A Systematic Review. PLoS Med 10(1): e1001 362. Doi: 10.1371/journal. p med.1001362

28. M. A. Chesney, J. R. Ickovics , D. B. Chambers , A. L. Gifford , J. Neidig , B. Zwickl , A. W. Wu & PATIENT CARE COMMITTEE & ADHERENCE WORKING GROUP OF THE OUTCOMES COMMITTEE OF THE ADULT AIDS CLINICAL TRIALS GROUP (AACTG) (2000): Self-reported adherence to antiretroviral medications among participants in HIV clinical trials: The AACTG Adherence Instruments, AIDS Care: Psychological and Socio-medical Aspects of AIDS/HIV, 12:3, 255-266

29. Hui-Chen Chu, Nai-Ying Ko (2013). Protocol for Using Mobile Phone Text Messaging to Improve Adherence to Highly Active Antiretroviral Therapy. Canadian Social Science, 9 (1), 92-96. Available from:http://www.cscanada.net/index.php/css/article/view/j.css.1923669720130901.1993
DOI: http://dx.doi.org/10.3968/j.css.1923669720130901.1993.

30. Rodriguez R, Shet A, Anthony J, Sidney K, Arumugam K, et al (2012). Supporting adherence to antiretroviral therapy with mobile phone reminders: Results from a cohort in South India. PLOS ONE 7(8): e40723. doi: 10.1371/journal.pone.0040723

31. Do N.T., Phiri K, Bussman H, Gaolathe T, Marlink R.G., Wester C.W: Psychosocial factors affecting medication adherence among HIV-infected adults receiving combination antiretroviral therapy (cART) in Botswana. AIDS Res. Human Retroviruses; 2010 June; 26(6): 685-691

32. Stacey M. Lavsa, Ashley Holzworth, Nicole T. Ansani: Selection of a validated scale for measuring medication adherence; Journal of American Pharmacists Association, 2011; 51(1): 90-94
