# Supplementary material for: Mobile phone reminders and peer counseling improve adherence and treatment outcomes of patients on ART in Malaysia: A randomized clinical trial
Source: PLoS One. 2017 May 16;12(5):e0177698. doi: 10.1371/journal.pone.0177698 (PMC5433794; doi:10.1371/journal.pone.0177698)
Supplement: S3 File — (PDF) [file pone.0177698.s003.pdf]

Malaysian Journal of Medicine and Health Sciences (ISSN 1675-8544); Vol. 11 (2) June 2015: 45-58

Baseline adherence, socio-demographic, clinical, immunological, virological and anthropometric characteristics of 242 HIV positive patients on ART in Malaysia

Abdulrahman Surajudeen Abiola,[1] Lekhraj Rampal*,[1] Norlijah Othman,[3] Faisal Ibrahim,[1] Hayati Kadir@Shahar,[1] Anuradha P. Radhakrishnan,[2]

[1]*Department of Community Health, Faculty of Medicine and Health Sciences, Universiti Putra Malaysia*
[2]*Hospital Sungai Buloh, Malaysia*
[3]*Department of Paediatrics, Faculty of Medicine and Health Sciences, Universiti Putra Malaysia*

ABSTRACT

Adherence to antiretroviral therapy (ART) prevents disease progression, and the emergence of resistant mutations. It also reduces morbidity, and the necessity for more frequent, complicated regimens which are also relatively more expensive. Minimum adherence levels of 95% are required for treatment success. Poor adherence to treatment remains a stumbling block to the success of treatment programs. This generates major concerns about possible resistance of the human immunodeficiency virus (HIV) to the currently available ARVs. This paper aims to describe baseline results from a cohort of 242 Malaysian patients receiving ART within the context of an intervention aimed to improve adherence and treatment outcomes among patients initiating ART.

A single-blinded Randomized Controlled Clinical Trial was conducted between January and December, 2014 in Hospital Sungai Buloh. Data on socio-demographic factors, clinical symptoms and adherence behavior of respondents was collected using modified, pre-validated

Adult AIDS Clinical Trials Group (AACTG) adherence questionnaires. Baseline CD4 count, viral load, weight, full blood count, blood pressure, Liver function and renal profile tests were also conducted and recorded. Data was analyzed using SPSS version 22 and R software.

Patients consisted of 215 (89%) males and 27 (11%) females. 117 (48%) were Malays, 98 (40%) were Chinese, 22 (9%) were Indians while 5 (2%) were of other ethnic minorities. The mean age for the intervention group was 32.1 ± 8.7 years while the mean age for the control group was 34.7 ± 9.5 years. Mean baseline adherence was 80.1 ± 19.6 and 85.1 ± 15.8 for the intervention and control groups respectively. Overall mean baseline CD4 count of patients was 222.97 ± 143.7 cells/mm^3 while overall mean viral load was 255237.85 ± 470618.9. Patients had a mean weight of 61.55 ± 11.0 kg and 61.47 ± 12.3 kg in the intervention and control groups, respectively.

Males account for about 90% of those initiating ART in the HIV clinic, at a relatively low CD4 count, high viral load and sub-optimal medication adherence levels at baseline.

Keywords: Adherence, Antiretroviral Therapy (ART), HIV/AIDS, CD4 count, Viral load, treatment outcomes.

INTRODUCTION

HIV was first diagnosed in Malaysia in 1986. By the end of 2013, there was a cumulative figure of 101,672 HIV cases reported to the Ministry of Health, 20,235 AIDS cases, and 16,340 deaths.[1] In 2002, the epidemic peaked with a rate of 28.5 per 100,000 population. Since then, there has been a steady decline achieved at a rate of 11.42 per 100,000 population in 2013.[1] By 2013, the number of persons living with HIV/AIDS increased to 86,324 with 3,393 new infections in the same year. The introduction of harm reduction programs since 2005 has resulted in a significant decline in the number of HIV infections through needle sharing. However, in recent years, there is increasing evidence that overlapping of injecting drug use (IDU) and risky sex behavior is occurring, resulting in increased HIV infection between the different populations. In 2011, sexual transmission had superseded IDU as the key driving factor of the epidemic with a ratio of 6 sexual transmissions for every 4 IDU reported.[2] There is a significant changing trend in HIV by gender with a male: female ratio of 9.6:1 in 2000 to 3.7:1 by 2013.[1] The incidence rate of HIV infection among adults 15-49 years old has decreased from 49% to 26% between 2001 and 2011.[3] Malaysia with an overall HIV prevalence of 0.5% and a concentrated epidemic, has about 17,369 patients as at the end of 2013[1] for which the Malaysian Government currently almost entirely provides all the funds for HIV treatment care and support for, at no

*Corresponding author: Prof. Dr. Lekhraj Rampal
dr_rampal1@hotmail.com / lekhraj@upm.edu.my

cost to the patients on first line medications and heavily subsidized for those on second line antiretrovirals (ARVs). However, the unmet need for Antiretroviral Therapy (ART) is estimated to be 50 – 60%. Despite these efforts, the globally recognized issues of injecting drug use, stigma and discrimination as well as poor adherence to treatment (clinic visits and medication adherence) remains a stumbling block to the success of treatment programs and generates major concerns about resistance of the HIV virus to the currently available ARVs.

Adherence to ARVs prevents disease progression, and the emergence of resistant mutations, thereby reducing morbidity, and the necessity for more frequent, complicated regimens which are also relatively more expensive. According to WHO (2005), minimum adherence levels of 95% are required for treatment success.[4-7] Other studies have demonstrated that ARV medication adherence levels of 54 – 95% is required to maintain prolonged viral load suppression depending on the allowable flexibility margins of each ART program. It is however generally accepted that those patients who adhere strictly to their medication achieve viral suppression, while those who are not adherent may not.[5-8] Poor adherence is a significant cause of treatment failure, disease progression and death among HIV patients. Once treatment failure results from poor adherence, the preventive opportunity that antiretroviral treatment provides is lost. Poor adherence also has grave socioeconomic impact on program funding, as more patients who fail on first line regimens have to be provided with the more expensive and complex second line medications.[3,9] Several studies have identified stigma and discrimination, pill burden, disclosure issues, depression, medication side effects, drug and alcohol use, unemployment, health and religious beliefs, low family income, lack of community support and integration, poor pre-initiation adherence counselling as some of the factors that contribute to poor medication adherence and the consequent rate of default and lost to follow up. Recent innovations using mobile phone technologies such as text messaging to improve medication adherence among patients on ART have been examined and implemented across many countries of the world including Kenya, Peru, Brazil, Botswana, USA with high quality evidence proving the efficacy of weekly SMS reminders to patients in improving adherence to ART when compared to standard care, particularly in 3 Randomized Controlled Trials (RCTs) done in Kenya.[10-12]

Another study systematically reviewed the scope and effectiveness of phone messaging for HIV/AIDS care based on different study designs (RCTs, Intervention studies using other study designs, qualitative and cross-sectional surveys) across US, UK, Kenya, Uganda, Botswana, South Africa, Peru, Pakistan among other countries and concluded that mobile phone messaging could play an important role in HIV/AIDS care and its use is acceptable.[13] However, further studies across low, middle and high income countries, scale-up of program evidence in hospitals, including cost-effectiveness analysis were recommended.

This paper describes baseline characteristics of a cohort of 242 Malaysian patients involved in a randomized controlled trial. The overall aim of the study was to investigate whether the introduction of mobile phone technology (SMS and telephone call reminders) can significantly improve adherence and treatment outcomes among HIV patients on ART in Malaysia. The study's main aim is in line with key activity 6 of strategy 2 in the current drive by Malaysian Government to improve adherence to treatment and detection of treatment failure.[3]

METHODS AND MATERIALS

A single-blinded Randomized Controlled Clinical Trial was conducted between January and December, 2014 in Hospital Sungai Buloh. It is the largest infectious disease hospital and one the foremost reference hospitals in Malaysia. It has over 6,000 HIV/AIDS patients on treatment and care, accounting for 35 – 40% of about 17,000+ patients currently on Highly Active Antiretroviral Therapy (HAART) in various centres across Malaysia. The centre provides a wide range of HIV/AIDS prevention, care and treatment services including diagnostics, pharmaceutical, clinical and support services to an average of 1000 HIV-positive patients per year, out of which an average of 400 - 500 patients are initiated on HAART yearly based on eligibility criteria. It maintains a high standard and quality of care based on international best practices and guidelines. It also provides training and research services including continuous medical education, specialist training and knowledge management.

Sample size calculation was done using the formula for calculating sample size in hypothesis testing by comparing two means (Lameshow et al., 1990)[14] which returned a sample size (n) maximized at 121 per group. Therefore a total sample size of 242 was applied for this study. Patients who were assessed and found to be eligible for ART commencement based on 2013 WHO guidelines, and with valid telephone numbers and able to read text messages were included in the study and randomized to either of two treatment arms based on simple, complete randomization technique in a ratio 1:1 for intervention and control groups, respectively. Written allocation of assignment was sealed in individual opaque envelopes marked with study identification numbers which was made available in the study clinic to allocate the target number of participants. After consenting to participate (by filling and signing a consent form) and meeting the inclusion criteria, screened subjects were enrolled and immediately afterwards were assigned to a randomized study arm by the study coordinator opening the sealed envelopes to determine allocation. Random

allocation considered and eliminated all forms of possible bias based on the socio-demographic characteristics of the clients. Baseline data on socio-demographic factors, clinical symptoms including TB status and Opportunistic Infection (OI) index, and adherence behaviour (over 2-4 weeks of adherence preparation and vitamin training) of respondents was collected using modified and pre-validated Adults AIDS Clinical Trial Group (AACTG) adherence questionnaires. Baseline CD4 count, viral load, weight, full blood count, blood pressure, Liver function and renal profile tests were also conducted and recorded. A "Reminder module" which included standardized weekly SMS medication reminders (sent at 9am every Monday); SMS reminder 3 days prior to scheduled clinic appointments (individualized and sent at lunch time), and an average of 90 second lunch hour telephone call reminders a day prior to scheduled clinic appointment (in addition to standard care - routine adherence counselling) was delivered consistently for 24 weeks to respondents in the intervention group by 2 trained PLHIV (research assistants) while respondents in the control group received standard care only. Each patient in the intervention group had a minimum of three (during clinic visits at month 1, month 3 and month 6) individual counselling sessions with the research assistants lasting an average of 15 minutes per encounter. To ensure confidentiality, typical medication reminder text messages included a short slogan in Malay language "Apa khabar" "Ini untuk menberithau anda ubat" meaning "How are you?" "This is to remind you of your medications". Appointment reminder text message was "Apa khabar" "Tolong ingat tarikh temu janji lusa" meaning "How are you?" "Remember your appointment day after tomorrow" and telephone conversation was standardized and short, with the message "Apa khabar" "Tolong ingat tarikh temu janji besok" meaning "How are you?" "Remember your appointment tomorrow". Patients were not required to provide any responses to the text messages. However, a log of text message communications and telephone calls was recorded and kept.

Adherence measurement was repeated at 3 and 6 months follow up using specialized AACTG follow up adherence questionnaires while CD4 count, viral load, weight, full blood count and blood pressure measurements were repeated at 6 months follow up period. Data was entered, cleaned and analyzed using SPSS version 22 and R software. Descriptive continuous data were presented as means ± standard deviation (SD), while categorical variables are presented as counts and percentages. Chi-square tests of independence, Fisher's exact test, ANOVA and student t-test for independent samples were conducted on the data. The differences were considered to be significant at $P<.05$.

Written approval for conducting the study was obtained from Universiti Putra Malaysia Ethics committee for Human Research and the Malaysian Ministry of Health's Institutional Review and Ethics Committee, prior to the commencement of the study. Written permission for conducting the study was obtained from the Medical Director of the hospital to use their clients.

RESULTS

Table 1 indicated that out of the 242 patients in the sample, 215 (88.8%) of them were males and 27 (11.2%) were females. Malays constituted the overall majority (n = 117, 48.3%) of respondents in the sample, 40.5% were Chinese (n = 98), 9.1% were Indians (n = 22) and 2.1% (n = 5) were of other minority ethnic origin such as Ibans. The overall mean age of respondents in the sample was 33.4 ± 9.2 SD (95% CI: 32.23 – 34.56) years and ranged from 18 to 64 years. The mean age for males was 33.1 ± 8.7 SD (95% CI: 31.93 – 34.27) years, while the mean age for females was 35.7 ± 12.3 SD (95% CI: 30.82 – 40.59). The income distribution of respondents showed that an overall majority of respondents (n = 188, 73.6%) earned RM 3499 and below, while 26.4% earned above RM 3500. Majority (n = 170, 70.2%) of the respondents attained educational level of less than bachelor degree while only 74 (29.7%) attained bachelor degree and above. Overall, majority (59.9%) of respondents were private organization employees, 14.9% were self- employed, and 12.8% were unemployed. Government employees were of relative minority (n = 21, 8.7%) while only 9 respondents (3.7%) reported other forms of employment such as students. Slightly over half (52.9%) of the respondents lived in urban location, 36% in semi-urban and 11.2% in rural location.

Females had lower (75.19 ± 24.8) mean baseline adherence compared to males (83.77 ± 17.2). However, this difference did not reach statistical significance ($t = 1.354$, df =240, P=.19). Overall, mean baseline adherence did not differ significantly by age group ($F(4, 237) = 0.425$, P=.79), ethnicity ($F(3, 238) = 0.150$, P=.93), income distribution ($F(4, 237) = 0.156$, P=.96), education level ($t = -1.173$, df =240, P=.24) and employment status ($F(4, 237) = 1.144$, P=.33). Patients who resided in semi-urban areas had significantly higher mean baseline adherence (90.90 ± 8.9) compared to those who resided in urban (80.25 ± 19.7) and rural (68.93 ± 32.0) locations.

Table 1: Baseline adherence rate by sociodemographic factors

Factors	n (%)	Mean baseline adherence	P value
**Age category**			
29yrs and below	102 (42.1)	81.04 ± 18.8	0.790□
30 – 39yrs	84 (34.7)	84.54 ± 16.0	
40 – 49yrs	42 (17.4)	81.55 ± 18.4	
50 -59yrs	11 (4.5)	90.73 ± 9.4	
60yrs and above	3 (1.2)	83.67 ± 16.3	
**Gender**			
Male	215 (88.8)	83.77 ± 17.2	0.185[a]
Female	27 (11.1)	75.19 ± 24.8	
**Ethnicity**			
Malay	117 (48.3)	82.99 ± 18.1	0.930□
Chinese	98 (40.5)	83.47 ± 17.0	
Indian	22 (9.1)	80.23 ± 19.7	
Others	5 (2.1)	77.20 ± 23.2	
**Education level**			
Bachelor degree and above	72 (29.7)	85.89 ± 14.1	0.243[a]
Less than Bachelor degree	170 (70.2)	81.51 ± 18.6	
**Monthly Income (RM)**			
<1500	60 (24.8)	82.20 ± 18.3	0.960□
1500 – 2499	57 (23.6)	83.49 ± 16.7	
2500 – 3499	61 (25.2)	80.90 ± 20.1	
3500 – 4999	30 (12.4)	84.17 ± 15.9	
>5000	34 (14.0)	85.00 ± 14. 9	
**Employment Status**			
Unemployed	31 (12.8)	81.65 ± 18.9	0.337□
Self-employed	36 (14.9)	86.78 ± 13.7	
Government employee	21 (8.7)	71.43 ± 28.7	
Private organization employee	145 (59.9)	83.49 ± 17.1	
Others	9 (3.7)	86.67 ± 13.3	
**Residential Location**			
Rural	27 (11.2)	68.93 ± 32.0	0.003□*
Semi-urban	87 (35.9)	90.90 ± 8.9	
Urban	128 (52.9)	80.25 ± 19.7	

Values are mean ± SD
[a]Value obtained by student t-test for independent samples.
□Value obtained by one-way ANOVA test.
*Significant at P<0.05

As shown in Table 2a, the male-to-female ratio of respondents in both treatment arms was fairly equal and showed no statistically significant difference. There were more Malays than Chinese in the intervention group while the reverse was observed in the control group. More Indians and other ethnic minorities were observed in the intervention group. Respondents in the intervention group were on average about two and a half years younger than those in the control group (t = -2.176, df =240, *P*=.03). There was no significant difference in income distribution (χ^2 = 8.647, df = 4, *P*=.07), employment distribution (χ^2 = 3.534, df = 4, *P*=.47) and residential location (χ^2 = 1.219, df = 2, *P*=.54) between respondents in the intervention and control groups as indicated in Table 2b.

Table 2a: Baseline socio-demographic characteristics of respondents by treatment group

Factors	Intervention group (n = 121)	Control group (n = 121)	P value
**Gender**			
Male	107 (88.4)	108 (89.3)	0.838[a]
Female	14 (11.6)	14 (10.7)	
**Ethnicity**			
Malay	64 (52.9)	53 (43.8)	0.036□
Chinese	39 (32.2)	59 (48.8)	
Indian	14 (11.6)	8 (6.6)	
Others	4 (3.3)	1 (0.8)	
**Age (yrs)**	32.1 ± 8.7	34.7 ± 9.5	0.031□
**Education level**			
Less than Bachelor degree	71 (58.7)	93 (76.9)	0.004[a]
Bachelor degree and above	50 (41.3)	28 (23.1)	
**Monthly Income (RM)**			
<1500	21 (17.4)	39 (32.2)	0.071[a]
1500 – 2499	29 (24.0)	28 (23.1)	
2500 – 3499	32 (26.4)	29 (24.0)	
3500 – 4999	18 (14.8)	12 (9.9)	
>5000	21 (17.4)	13 (10.7)	

Values are n (%) or mean ± SD.
[a]Value obtained by chi-square test.
□Value obtained by Fisher's exact test.
□Value obtained by student t- test for independent samples. RM = Ringgit Malaysia

Table 2b: Baseline socio-demographic characteristics of respondents by treatment group

Factors	Intervention group (n = 121)	Control group (n = 121)	P value
**Employment Status**			
Unemployed	13 (10.7)	18 (14.9)	0.473[a]
Self-employed	19 (15.7)	17 (14.0)	
Government employee	14 (11.6)	7 (5.8)	
Private organization employee	70 (57.9)	75 (61.9)	
Others	5 (4.1)	4 (3.3)	
**Residential Location**			
Rural	11 (9.1)	16 (13.2)	0.544[a]
Semi-urban	43 (35.5)	44 (36.4)	
Urban	67 (55.4)	61 (50.4)	

Values are n (%) or mean ± SD.
[a]Value obtained by chi-square test.

Table 3a indicated that overall, 47.9% of respondents were placed on ZDV/3TC/EFV regimen, 47.1% took TDF/FTC/EFV regimen at baseline (ART initiation) while other HAART regimens accounted for only 5% of the distribution altogether. A similar distribution was observed among respondents in both intervention and control group as approximately 95% of respondents were placed on either ZDV/3TC/EFV or TDF/FTC/EFV regimen in each group.

A 30 day recent history of alcohol use was assessed among respondents. Overall, 195 (80%) out of a total of 242 patients in the sample never had a drink containing alcohol while the remaining 20% had alcohol at various periods and quantities over the last 30 days. 10% of respondents had alcohol once a month while only 1 respondent in the control group had alcohol daily. There was no statistically significant difference in history of alcohol use between respondents in the intervention and control group (Fisher's exact *P*=1.00).

Previous history of drug use (IDUs and non-IDUs) and frequency of usage over the past 6 months was assessed among respondents. As highlighted in Tables 3a and 4, only about 6% of respondents have ever used any recreational drug before. 234 (97%) out of a total 242 respondents in the sample never used marijuana, 239 (99%) never used cocaine, 237 (98%) never used heroine or opioids, 227 (94%) never used amphetamines in the past while only 3 (1%) patients were currently on methadone treatment. This pattern was same as observed in the drug use history of respondents in both treatment arms, hence, no statistically significant group difference was observed for any of the above mentioned drugs in the past.

Based on the information on when a patient last missed their medications and the weighted average of the number of doses missed at each time period, participants were further categorized into Good, Fair or Poor adherence levels. As indicated in Table 3b, overall, 194 (80%) respondents in the sample had Good adherence while the remaining 20% had poor adherence. Among the intervention group, about 77% of respondents had good adherence as compared to 84% in the control group. There was no significant difference in the baseline medication adherence levels of respondents in the intervention and control groups (χ^2 = 1.663, df = 1, *P*=.19).

The overall mean baseline medication adherence was 82.6 ± 17.7 SD for the sample. There was no significant difference in the mean baseline medication adherence (80.1 ± 19.6 SD) for the intervention group as compared to the control group (85.1 ± 15.8 SD) (t = -1.252, df = 235.6, *P*=.21).

It is also explicit from Table 3b that overall, majority (80%) of respondents had disclosed their HIV status to a family or close friend, while 36% had at least one comorbidity at baseline. Sexual transmission was the most likely mode of HIV transmission in about 91% of respondents who initiated HAART in the current study, followed by other modes of transmission predominantly IDUs and blood transfusion accounting for 5%, while about 4% of respondents didn't know the most likely source of infection. Results from the current study indicated that 37% of respondents were heterosexuals while 54% were homosexuals and 9% were bisexual.

About 12% higher proportion of respondents in the control group had disclosed their HIV status to a family or close friend (χ^2 = 5.094, df = 1, *P*=.02), and reported more comorbidity at baseline (χ^2 = 8.730, df = 1, *P*<.01) than the intervention group. Although there were slightly more homosexuals in the intervention group than the control group, this difference did not reach statistical significance (χ^2 =4.030, df = 1, *P*=.26).

In terms of TB status at baseline, overall only about 18% of respondents were either currently on TB treatment (4%) or suspected clinically for TB (14%). 83% of respondents had no signs or symptoms of any new OI, 9% were Hepatitis B positive while 7% were Hepatitis C positive. There was no statistically significant difference in TB status (χ^2 = 1.731, df = 2, *P*=.42), OI index (Fisher's exact *P*=.28), Hepatitis B (χ^2 = 1.800, df = 1, *P*=.18) and Hepatitis C status (χ^2 = 1.071, df = 1, *P*=.30) between respondents in the intervention group and those in the control group.

Table 3a: Baseline therapy-related and patient-related factors by treatment group

Factors	Intervention group (n = 121)	Control group (n = 121)	P value
**Therapy-related factors**			
HAART Regimen			
ZDV/3TC/NVP	2 (1.7)	5 (4.1)	1.000[a]
ZDV/3TC/EFV	59 (48.8)	57 (47.1)	
TDF/FTC/NVP	1 (0.8)	1 (0.8)	
TDF/FTC/EFV	58 (47.9)	56 (46.3)	
ZDV/3TC/RALTEGRAVIR	1 (0.8)	1 (0.8)	
D4T/3TC/EFV	0 (0.0)	1 (0.8)	
**Patient-related factors**			

Alcohol use (past 30 days)			
Never	100 (82.6)	95 (78.5)	1.000[a]
Once a month	12 (9.9)	13 (10.7)	
2 or 3 times a month	6 (5.0)	7 (5.8)	
3 or 4 times a week	3 (2.5)	5 (4.1)	
Daily	0 (0.0)	1 (0.8)	
Drug use history			
(a) Marijuana			
No	117 (96.7)	117 (96.7)	0.639[a]
Yes	4 (3.3)	4 (3.3)	
(b) Cocaine			
No	119 (98.3)	120 (99.2)	0.500[a]
Yes	2 (1.7)	1 (0.8)	
(c) Heroine			
No	120 (99.2)	117 (96.7)	0.185[a]
Yes	1 (0.8)	4 (3.3)	
(d) Amphetamines			
No	115 (95.0)	112 (92.6)	0.424□
Yes	6 (5.0)	9 (7.4)	
(e) Opioids			
No	118 (97.5)	119 (98.3)	0.500[a]
Yes	3 (2.5)	2 (1.7)	
(f) Methadone treatment			
No	119 (98.3)	120 (99.2)	0.500[a]
Yes	2 (1.7)	1 (0.8)	

Values are n (%) or mean ± SD.
[a]Value obtained by Fisher's exact test.
□Value obtained by chi-square test.
□Value obtained by student t- test for independent samples.

Table 3b: Baseline medication adherence, condition and treatment-related factors by treatment group

Factors	Intervention group (n = 121)	Control group (n = 121)	P value
**Treatment-related factors**			
When patient last missed medications			
Not at all	93 (76.9)	101 (83.5)	0.745[a]
1-3 months ago	1 (0.8)	1 (0.8)	
2-4 weeks ago	4 (3.3)	2 (1.7)	
1-2 weeks ago	6 (5.0)	5 (4.1)	
Within the past week	17 (14.0)	12 (9.9)	
**Mean adherence**	80.1 ± 19.6	85.1 ± 15.8	0.212□
**Baseline medication adherence level**			
Poor adherence	28 (23.1)	20 (16.5)	0.197□
Good adherence	93 (76.9)	101 (83.5)	

**Condition-related factors**			
**Disclosure status**			
No	31 (25.6)	17 (14.0)	0.024[a]
Yes	90 (74.4)	104 (86.0)	
**Most likely source of HIV infection**			
Don't know	4 (3.3)	6 (5.0)	0.158[a]
Others (IVDU, blood transfusion)	3 (2.5)	9 (7.4)	
Sexual transmission	114 (94.2)	106 (87.6)	
**TB Status**			
No signs and symptoms of TB	103 (85.1)	96 (79.3)	0.421[a]
TB suspect	13 (10.7)	20 (16.5)	
Currently on TB treatment	5 (4.1)	5 (4.1)	
**OI Index**			
No signs & symptoms of any new OI	103 (85.1)	97 (80.2)	0.283□
1 or more WHO stage 2 defining disease	1 (0.8)	1 (0.8)	
1 or more WHO stage 3 defining disease	8 (6.6)	5 (4.1)	
1 or more WHO stage 4 defining disease	9 (7.4)	18 (14.9)	
**Hepatitis B status**			
Negative	107 (88.4)	113 (93.4)	0.180[a]
Positive	14 (11.6)	8 (6.6)	
**Hepatitis C status**			
Negative	115 (95.0)	111 (91.7)	0.301[a]
Positive	6 (5.0)	10 (8.3)	

Values are n (%) or mean ± SD.
[a]Value obtained by Fisher's exact test.
□Value obtained by chi-square test.
□Value obtained by student t- test for independent samples.

Table 4 also indicated that mean baseline adherence did not differ significantly by respondents' source of HIV infection (F (2, 239) = 0.448, *P*=.64), sexual orientation (F (2, 239) = 0.309, *P*=.82), disclosure status (t (240) = 1.131, *P*=.26), alcohol (F (4, 237) = 1.134, *P*=.34) and drug use history.

Notably, patients who acquired HIV infection through Intravenous Drug Use and blood transfusion had lowest mean baseline adherence of 77.08 ± 12.9. Whereas there were more homosexuals than heterosexuals and bisexuals in the sample, mean baseline adherence was fairly similar among these three groups. A higher mean baseline adherence (83.82 ± 16.1) was observed among respondents who had disclosed their HIV status to a friend or family member, as compared to those who had not. However, this difference did not reach statistical significance.

Interestingly, and perhaps expectedly, an optimal (>95%) and highest mean baseline adherence was observed among respondents who were currently on TB treatment as compared to those who were TB suspects (90.79 ± 9.2) or patients who had no signs and symptoms of TB (80.83 ± 19.1). This difference was however not statistically significant.

We observed that mean baseline adherence was higher among patients who used alcohol more frequently, although this difference did not reach statistical significance. With the exception of 13 patients who used opioids and marijuana, we found a sub-optimal and similar mean baseline adherence of 77.41 ± 22.5 for patients on methadone replacement and positive history of cocaine, heroine and amphetamine use.

Table 4: Baseline adherence rate by patient-related and condition-related factors

Factors	n (%)	Mean baseline adherence	P value
**Most likely source of HIV infection**			
Don't know	10 (4.1)	88.30 ± 12.2	0.640□
Others (IVDU, blood transfusion)	12 (4.9)	77.08 ± 12.9	
Sexual transmission	220 (90.9)	82.88 ± 17.3	
**Sexual orientation**			
Homosexual	131 (54.1)	82.59 ± 18.0	0.819□
Heterosexual	89 (36.8)	83.30 ± 16.8	
Bisexual	22 (9.1)	82.69 ± 17.3	
Transgender	0 (0.0)	-	
**Disclosure status**			
No	48 (19.8)	78.75 ± 21.2	0.259[a]
Yes	194 (80.1)	83.82 ± 16.1	
**TB Status**			
No signs and symptoms of TB	199 (82.2)	80.83 ± 19.1	0.050□
TB suspect	33 (13.6)	90.79 ± 9.2	
Currently on TB treatment	10 (4.1)	96.00 ± 4.1	
**History of alcohol use (past 30 days)**			
Never	195 (80.6)	84.22 ± 16.3	0.341□
Once a month	25 (10.3)	76.20 ± 13.8	
2 or 3 times a month	13 (5.4)	71.23 ± 28.9	
3 or 4 times a week	8 (3.3)	86.38 ± 13.6	
Daily	1 (0.4)	96.00	
**Drug use history**			
(a) Marijuana			
No	234 (96.7)	82.85 ± 17.1	0.902[a]
Yes	8 (3.3)	81.63 ± 18.3	
(b) Cocaine			
No	239 (98.8)	82.89 ± 17.3	0.716[a]
Yes	3 (1.2)	77.00 ± 22.9	
(c) Heroine			
No	237 (97.9)	82.92 ± 17.5	0.685[a]
Yes	5 (2.1)	77.80 ± 22.1	
(d) Amphetamines			
No	227 (93.8)	83.14 ± 16.8	0.478[a]
Yes	15 (6.2)	77.87 ± 22.1	
(e) Opioids			
No	237 (97.9)	82.54 ± 18.0	0.285[a]
Yes	5 (2.1)	96.00 ± 3.9	
(f) Methadone treatment			
No	239 (98.8)	82.89 ± 18.1	0.716[a]
Yes	3 (1.2)	77.00 ± 22.9	

Values are mean ± SD. [a]Value obtained by student t-test for independent samples; □one-way ANOVA.

Table 5 below showed that the overall mean CD4 cell count for the sample was 222.98 ± 143.7 SD. The mean CD4 count of respondents in the intervention group was 232.64 ± 137.9 SD and was not significantly higher than that of the control group (213.31 ± 149.4 SD) (t = 1.046, df = 240, *P*=.29).

Mean viral load log for the sample was 4.73 ± 1.0 SD. In the intervention group, the mean viral load log was 4.61 ± 0.9 which was slightly lower than that of the control group (4.85 ± 1.1 SD). There was no significant difference in the mean viral load log between respondents in the intervention and control groups (t = -1.465, df = 240, *P*=.14).

The overall mean weight of respondents at baseline was 61.33 ± 11.9 SD. There was no significant difference between the mean weight of respondents in the intervention group (61.19 ± 11.4 SD) compared to that of respondents in the control group (61.47 ± 12.3 SD) (t = 0.056, df = 240, *P*=.96).

Table 5: Baseline CD4 count, Viral Load and weight of respondents

Factors	Intervention group (n = 121)	Control group (n = 121)	P value
CD4 count (cells/µl)	232.64 ± 137.9	213.31 ± 149.4	0.297
Viral Load (Log10)	4.61 ± 0.9	4.85 ± 1.1	0.144
Weight (Kg)	61.19 ± 11.4	61.47 ± 12.3	0.955

Values are mean ± SD.
Values obtained by student t- test for independent samples.

DISCUSSION

The mean age (in years) of 242 respondents was 33.4 ± 9.2 SD (58% were of the age group 30-39 years) which is similar to the findings of previous studies in Malaysia,[1,2] Kenya[11] and Brazil.[15] According to WHO, poverty is a significant determinant of disease occurrence. Very few studies have identified poor financial incentives that include out-of-pocket expenses and high transportation costs from frequent hospital visits as some of the factors that contribute to non-adherence. These may explain why patients from rural areas had poorer adherence as shown in this study. Thrice as many respondents in the current study as in a similar study in Kelantan, Malaysia earned RM 1,500 and above. The reverse was true for those who earned below RM 1,500. This is perhaps due to geographical variation in earning capacity and cost of living between residents of Kelantan and Selangor, Malaysia which may potentially affect adherence. Despite this distribution, we found that among patients initiating ART in Selangor, mean adherence at baseline was not dependent on patient's income status.

We found about five times as many persons in Selangor as Kelantan[16] attended tertiary education and also had higher mean baseline adherence. This population of PLHIV graduates is also 43% higher than what was reported elsewhere in Botswana.[17] State-specific socio-economic and cultural dynamics which influence access and affordability of tertiary education between and among populations, may explain these variations. However, our study as above also showed that educational level does not have significant effect on baseline adherence.

The correlation between employment status, available monthly income (to cover transportation costs for clinic follow up visits, adequate nutrition and any incidental out-of-pocket expenses) and adherence to treatment (regular clinic follow up visits and strict medication adherence) has been widely reported in literature. Our study found that 83% of respondents in the sample were employed (either self-employed, Government or private organization employees) while 17% were unemployed. These findings represent much higher values than that reported in Kelantan but not Bostwana. However, our study also showed that baseline adherence is not significantly affected by patient's employment status. Exploring the interplay of socio-economic and other developmental factors that influence job availability and income distribution becomes a useful planning tool for health interventions.

A similar intervention study in Kenya[11] reported higher proportion of urban dwellers in the sample than the current study which also had 36% semi-urban dwellers. Interestingly, we found in this study, a significantly higher mean baseline adherence among semi-urban dwellers than urban and rural dwellers. While poor access to information and low education level may perhaps explain why rural dwellers had the poorest adherence in this study, it appears that targeted, integrated health interventions to address low income, access to information, education and qualitative adherence counselling among rural dwellers would be most beneficial.

To the best of our knowledge, this is the first study to assess and report medication adherence among HIV positive patients receiving ART in Malaysia. Our study utilized a standardized, modified AACTG adherence questionnaire[18] to measure medication adherence by self-report. The overall mean adherence at baseline was 82.6 ± 17.7 SD. This is not only consistent with previous evidence from studies with varying designs from rural Uganda[19] to Urban United States[20] or even China[21] but also provides new evidence in a concentrated epidemic like Malaysia. Much fewer studies in low-to-middle income countries, predominantly observational, have reported higher baseline mean adherence than the current study.[22,23] Apart from differences in socio-demographic characteristics of respondents which may explain the observed similarities and/or differences in results between our study and those highlighted above, there was also significant difference in the study population in terms of duration on ART treatment. While our study utilized ART-naïve patients at baseline, both Ugandan studies above recruited patients who had been on ART for a period between 0-3 years.

Decision-making by HIV clinicians on when to initiate ART among HIV positive patients is usually based on clinical (WHO staging) and immunological parameters (CD4 count) of the patients at baseline. According to WHO (2013),[24] ART should be initiated among HIV positive patients in WHO clinical stage 1 and 2 whose CD4 count is ≤500 cells/mm³ (CD4 ≤350 cells/mm³ as a priority). It also recommended ART commencement among patients with severe or advanced HIV disease (WHO clinical stage 3 and 4) irrespective of their CD4 count. In the current study, the overall mean CD4 count of respondents at baseline was 222.98 ± 143.7 SD. No significant group differences were observed. Much higher figures have been previously reported in intervention studies in United States[25] and China[21] but not in African studies[11,17] where majority of patients appear to initiate ART at generally lower CD4 count due to higher burden and limited access to ART. As evidence continues to influence practice (EBM), it will not be surprising to see newer studies reporting higher pre-initiation CD4 count based on 2013 WHO guidelines than earlier studies conducted based on 2010 WHO guidelines.

Although results of viral load test at baseline are not critical to decision-making on when to initiate ART, the value of viral load monitoring in diagnosing treatment failure and deciding when to switch to second-line ART or salvage therapy cannot be over-emphasized. HIV clinicians where feasible, have however measured and compared baseline viral load test results with those repeated after 4-6 months of antiretroviral therapy in deciding whether a patient has achieved viral suppression or not. In the clinical setting where the current research was conducted, it is routine practice for clinicians to order for viral load test among other investigations at baseline, for every HIV positive patient commencing ART. Typical viral load test results in this setting are reported as absolute viral load counts per mL of blood as well as log10 copies per mL of blood. The overall mean viral load count in the current study was 252662.74 ± 528133.95 SD. Twice higher pre-initiation viral load was previously reported in United States[25] than in Malaysia, Botswana[17] and Kenya.[11] Perhaps, differences in clinical stage and biomedical parameters of patients at initiation of ART may explain these variations.

We found a much higher mean weight of respondents at baseline (61.33 ± 11.9 SD) kg than a similar study in rural Rwanda,[26] and although BMI measurement was beyond the scope of the current study, values lower than Malaysian national average were reported in Tanzania[27] and Kenya.[28] No significant group differences in weight distribution of respondents was found in our study. Differences in the respondents' clinical stage at presentation may explain this variations.

With the implementation of WHO STOP-TB strategy and integration of TB/HIV services in the clinical setting in which our study was conducted, an improved TB case detection rate was not unexpected. In the current study, overall, only about 18% of respondents were either currently on TB treatment (4%) or suspected clinically for TB (14%) while 82% had no signs and symptoms of TB at baseline. Our study found a TB-HIV co-infection rate of 4.1% among respondents in each of the intervention and control groups. These results although slightly lower, are consistent with the 2013 TB-HIV co-infection rate of 6.1% reported by 2014 Malaysia Global AIDS Response Country Progress Report [1] and from a clinical setting in India[29] of 4.85%. As much as four to ten-fold higher TB-HIV co-infection rates have been reported in rural Rwanda[26] and among TB suspects in Kenya,[30] respectively. Relative differences in National programme focus as well as level of availability and access to TB-HIV diagnostic services may explain the observed continental variations.

We found optimal (>95%) baseline adherence among all the TB-HIV co-infected patients in our sample compared to previous similar studies reporting up to 56% non-adherence among co-infected patients in other developing countries such as Uganda, Nigeria and Ethiopia.[31 - 33]

In the setting of the current study, respondents in the sample were assessed for opportunistic infections at baseline and during each follow up visit. Overall, about 4 in 5 respondents were free of any OIs at baseline. Same was the case in both treatment arms. These results are similar to the findings of a similar study conducted in Tanzania[27] in which about 24% of respondents had one or more symptoms and signs of WHO stage 2, stage 3 or stage 4 defining diseases at baseline. Due to enormity of possible opportunistic infections among HIV positive patients, it is often difficult for studies to track and report effectively on these conditions, hence the paucity of data on OI index of patients at baseline, in literature.

This study has some important limitations. In addition to the fact that self-report of adherence behaviour is widely believed to over-estimate true adherence, baseline adherence scores presented in this study might have been further exaggerated by estimating adherence behaviour based on 2 – 4 weeks of vitamin training (with little or no side effects) and adherence preparation prior to ART initiation, and not necessarily HAART.

CONCLUSION

Overall, the cohort of 242 patients involved in this trial were largely males (88.8%), with a mean age of 33.1 (95% CI: 31.93 – 34.27) years but higher mean baseline adherence than females. Semi-urban dwellers had higher baseline adherence than urban and rural dwellers. Mean baseline adherence of participants in the sample did not differ by other socio-demographic, patient or condition-related factors. However, significant group differences in the mean age, ethnic distribution and education level of participants was observed between treatment arms. On the average, this cohort initiated ART at suboptimal baseline adherence, low CD4 count and high viral load, with about 20% of patients needing adherence interventions from the outset, in addition to routine strategic and collaborative treatment adherence planning between the patient and the care-giver before commencement of and during the course of ART, in order to achieve successful treatment outcomes.

ACKNOWLEDGEMENTS

The authors wish to acknowledge the critical role and contribution of Mrs. Jayanthi Arumugam and the entire health care team of Infectious disease clinic, Hospital Sungai Buloh to the successful conduct of this research.

REFERENCES

1. Ministry of Health. Global AIDS Response Country Progress Report Malaysia 2014.

2. Ministry of Health. Global AIDS Response Country Progress Report Malaysia 2012.

3. Malaysia National Strategic Plan 2011 – 2015.

4. Fairly CK, Permana A, Read TRH. Long term utility of measuring adherence by self-report compared with pharmacy record in a routine clinic setting, HIV Medicine; 2005 (6): 366-369.

5. Jani AA. Adherence to HIV treatment regimens: recommendations for best practices, 2004, pp. 3-72. Available from http://www.alpha.org/ppp/hiv

6. Paterson DL, Swindells S, Mohr J, Brester M, Vergis EN, Squire C, Wagener MM, & Singh N. Adherence to protease inhibitor therapy and outcomes of patients with HIV infection. Annals of Internal Medicine; 2000, 133 (1): 21-30.

7. Saple D. Improving adherence to anti-retroviral therapy (Review articles). India Journal of Dermatology, Venereology and Leprology; 2005. Available from: http://www.highbeam.com/librarydoc3.asp?DOCID =1G1: 136743728&num=1&ao=&Fr (accessed 21.04.14).

8. Preininger L, Cantwell-McNelis K, James C, Sullivan MC, Szabo S, & Bincsik A. Long term Medication adherence in patients receiving antiretroviral therapy, Current HIV Research; 2011 (9): 253-255.

9. WHO (2007). 'Prioritizing Second-Line Anti-retroviral Drugs for Adults and Adolescents: A Public Health Approach'. Report of a WHO Working Group Meeting, 21-22 May, 2007.

10. Hovart T, Azman H, Kennedy GE, Rutherford GW. Mobile phone text messaging for promoting adherence to antiretroviral therapy in patients with HIV infection. Cochrane Database of Systematic Reviews; 2012, Issue 3, Art. No.: CD009756, DOI: 10.1002/14651858.CD009756.

11. Lester RT, Ritvo P, Mills EJ, Kariri A, Karanja S, Chung MH, Jack W., et al. Effects of a mobile phone short message service on antiretroviral treatment adherence in Kenya (WelTel Kenya 1): a randomized trial. *Lancet*; 2010, Nov. 27; 376 (9755): 1838-45.

12. Pop-Eleches C, Thirumurthy H, Habyarimana JP, Zivin JG, Goldstein MP, de Walqu D., et al. Mobile phone technologies improve adherence to antiretroviral treatment in a resource-limited setting: a randomized controlled trial of text message reminders. AIDS; 2011, 26(6): 825-34.

13. van Velthoven MHMMT, Brusamento S, Majeed A, & Car J. Scope and effectiveness phone messaging for HIV/AIDS care: A systematic review. Psychology, Health & Medicine; 18(2): 182-202.

14. Lemeshow, S., Hosmer, D. W., Klar, J., Lwanga, S. K and World Health Organization (1990). Adequacy of sample size in health studies. John wiley & sons Ltd.

15. Da Costa TM, Barbosa BJP, e Costa DAG, Sigulem D, Marin HD, Filho AC, Pisa IT. Results of a randomized controlled trial assess the effects of mobile SMS-based intervention on treatment adherence in HIV/AIDS-infected Brazilian women and impressions and satisfaction with respect to incoming messages. *International Journal of Medical Informatics*; 2012 (81): 257-269.

16. Hasanah CI, Zaliha AR, & Mahiran M. Factors influencing the quality of life in patients with HIV in Malaysia. Quality of Life Research, 2011, 20(1): 91-100.

17. Ndubuka NO, Ehlers VJ. Adult patients' adherence to antiretroviral treatment: a survey correlating pharmacy refill records and pill counts with immunological and virological indices. *International Journal of Nursing Studies*; 2011 (48): 1323-1329.

18. Chesney, M. A., Ickovics, J. R., Chambers, D. B., Gifford, A. L., Neidig, J., Zwickl, B., Wu, A.W. & Patient care committee & adherence working group of the outcomes committee of the Adults AIDS Clinical Trials Group (AACTG) (2000). Self-reported adherence to antiretroviral medications among participants in HIV clinical trials: The AACTG Adherence Instruments, AIDS Care: Psychological and Socio-medical Aspects of AIDS/HIV, 12:3, 255-266.

19. Kunutsor S, Walley J, Katabira E, Muchuro S, Balidawa H, Namagala E, & Ikoona E. Using mobile phones to improve clinic attendance amongst an antiretroviral treatment cohort in rural Uganda: A cross-sectional and prospective study. AIDS & Behaviour; 2010 (14): 1347-1353.

20. Hardy H, Kumar V, Doros G, Farmer E, Drainoni M-L, Rybin D, Skolnik PR. Randomized controlled trial of a personalized cellular phone reminder system to enhance adherence to antiretroviral therapy. AIDS Patient Care STDS; 2011 (25): 153-161.

21. Sabin LL, DeSilva MB, Hamer DH, Xu K, Zhang J, Li Tao, Wilson IB, & Gill CJ. Using Electronic Drug Monitor Feedback to Improve Adherence to Antiretroviral Therapy among HIV-Positive Patients in China. AIDS Behav; 2010 Jun; 14(3): 580-589. PMCID:PMC2865631

22. Kunutsor, S., Evans, M., Thoulass, J., Walley, J., Katabira, E., Newell, J. N., & Ikoona, E. (2010). Ascertaining baseline levels of antiretroviral therapy adherence in Uganda: a multimethod approach. *JAIDS Journal of Acquired Immune Deficiency Syndromes*, 55(2), 221-224.

23. Kunutsor, S., Walley, J., Katabira, E., Muchuro, S., Balidawa, H., Namagala, E., & Ikoona, E. (2010). Clinic attendance for medication refills and medication adherence amongst an antiretroviral treatment cohort in Uganda: a prospective study. AIDS research and treatment, 2010.

24. WHO (2013). 'Consolidated guidelines on the use of antiretroviral drugs for treating and preventing HIV infection, recommendations for a public health approach'.

25. Reynolds NR, Testa MA, Su M, Chesney MA, Neidig JL, Frank I, Smith S., et al. Telephone Support to Improve Antiretroviral Medication Adherence – A Multisite Randomized Controlled Trial. *Journal of Acquired Immune Deficiency Syndrome*; 2008, 47(1): 62-68.

26. Rich ML, Miller AC, Niyigena P, Franke MF, Niyonzima JB, Socci A, Drobac PC., et al. Excellent Clinical Outcomes and High Retention in Care Among Adults in a Community-Based HIV Treatment Program in Rural Rwanda. *JAIDS Journal of Acquired Immune Deficiency Syndromes*; 2012, 59(3): e35-e42. doi:10.1097/QAI.0b013e31824476c4

27. Mugusi F, Mugusi S, Bakari M, Hejdemann B, Josiah R, Janabi M, Aboud S., et al. Enhancing adherence to antiretroviral therapy at the HIV clinic in resource constrained countries; the Tanzanian experience. *Tropical Medicine and International Health*; 2009 (14): 1226-1232.

28. Sarna A, Luchters S, Geibel S, Chersich MF, Munyao P, Kaai S, & Rutenberg N. Short-and long-term efficacy of modified directly observed antiretroviral treatment in Mombasa, Kenya: a randomized trial. *JAIDS Journal of Acquired Immune Deficiency Syndromes*; 2008, 48(5), 611-619.

29. Dewan PK, Gupta D, Williams BG, Thakur R, Bacahani D, Khera A, Wares D., et al. National Estimate of HIV sero-prevalence among tuberculosis patients in India. *The International Journal of Tuberculosis and Lung Disease*; 2010, 14(2): 247-249(3).

30. Nyamogoba HDN, Mbuthia G, Mining S, Kikuvi G, Kikuvi R, Mpoke S, & Waiyaki PG. HIV co-infection with tuberculous and non-tuberculous mycobacteria in western Kenya: challenges in the diagnosis and management. African health sciences; 2013, 12(3), 305-311.

31. Amuha, M. G., Kutyabami, P., Kitutu, F. E., Odoi-Adome, R., & Kalyango, J. N. (2009). Non-adherence to anti-TB drugs among TB/HIV co-infected patients in Mbarara Hospital Uganda: Prevalence and associated factors. African Health Sciences, 9(Suppl 1), S8–S15.

32. Adebimpe, W. O., Asekun-Olarinmoye, E. O., Hassan, A. O., Abodunrin, O. L., Olarewaju, S., & Akindele, A. A. (2011). Treatment Outcomes among Human Immunodeficiency Virus and Tuberculosis Co-Infected Pregnant Women in Resource Poor Settings of South-western Nigeria. *Sierra Leone Journal of Biomedical Research*; 2011, 3(3), pp. 151-156.

33. Eticha, T., Kassa, E. (2014). Non-Adherence to Anti-TB Drugs and Its Predictors among TB/HIV Co-Infected Patients in Mekelle, Ethiopia. *J Bioanal Biomed*; 2014, 6: 061-064. doi:10.4172/1948-593X.1000113.
